# Cytotoxicity of crystals involves RIPK3-MLKL-mediated necroptosis

**DOI:** 10.1038/ncomms10274

**Published:** 2016-01-28

**Authors:** Shrikant R. Mulay, Jyaysi Desai, Santhosh V. Kumar, Jonathan N. Eberhard, Dana Thomasova, Simone Romoli, Melissa Grigorescu, Onkar P. Kulkarni, Bastian Popper, Volker Vielhauer, Gabriele Zuchtriegel, Christoph Reichel, Jan Hinrich Bräsen, Paola Romagnani, Rostyslav Bilyy, Luis E. Munoz, Martin Herrmann, Helen Liapis, Stefan Krautwald, Andreas Linkermann, Hans-Joachim Anders

**Affiliations:** 1 Medizinische Klinik und Poliklinik IV, Klinikum der Universität, München, Munich 80336, Germany; 2 Department of Anatomy and Cell Biology, Ludwig-Maximilians Universität, Munich 80336, Germany; 3 Department of Otorhinolaryngology, Head and Neck Surgery, University of Munich, Munich 81377, Germany; 4 Walter Brendel Center for Experimental Medicine, University of Munich, Munich 81377, Germany; 5 Department of Nephropathology, Institute for Pathology, Hannover Medical School, Hannover 30625, Germany; 6 Excellence Centre for Research, Transfer and High Education for the Development of De Novo Therapies (DENOTHE), University of Florence, Florence 50139, Italy; 7 Danylo Halytsky Lviv National Medical University, Lviv 79010, Ukraine; 8 Department for Internal Medicine, University Hospital Erlangen, Institute for Clinical Immunology, Erlangen 91054, Germany; 9 Department of Pathology and Immunology, Washington University School of Medicine, Saint Louis, Missouri 63110, USA; 10 Nephropath, Little Rock, Arkansas 72211, USA; 11 Division of Nephrology and Hypertension, Christian-Albrechts-University, Kiel 24105, Germany

## Abstract

Crystals cause injury in numerous disorders, and induce inflammation via the NLRP3 inflammasome, however, it remains unclear how crystals induce cell death. Here we report that crystals of calcium oxalate, monosodium urate, calcium pyrophosphate dihydrate and cystine trigger caspase-independent cell death in five different cell types, which is blocked by necrostatin-1. RNA interference for receptor-interacting protein kinase 3 (RIPK3) or mixed lineage kinase domain like (MLKL), two core proteins of the necroptosis pathway, blocks crystal cytotoxicity. Consistent with this, deficiency of RIPK3 or MLKL prevents oxalate crystal-induced acute kidney injury. The related tissue inflammation drives TNF-α-related necroptosis. Also in human oxalate crystal-related acute kidney injury, dying tubular cells stain positive for phosphorylated MLKL. Furthermore, necrostatin-1 and necrosulfonamide, an inhibitor for human MLKL suppress crystal-induced cell death in human renal progenitor cells. Together, TNF-α/TNFR1, RIPK1, RIPK3 and MLKL are molecular targets to limit crystal-induced cytotoxicity, tissue injury and organ failure.

## Abstract (web summary)

Kidney stone disease is caused by accumulation of oxalate crystals, which trigger tissue injury, inflammation and cell death. Mulay *et al*. show that crystals induce cell death in the kidney through necroptosis, and propose that this pathway may be a target for the treatment of crystal-induced disease.

## Graphical abstract

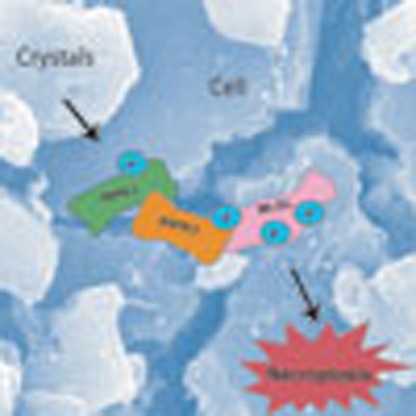

Crystals are deposits of various sizes and shapes composed of atoms, ions or biomolecules, frequently with tissue injury, inflammation and remodelling. Two mechanisms may explain this association: (I) nucleation or crystal growth from a seed crystal formed on a surface medium, for example tubular epithelial cells, urolithiasis forming at Randall's plaques, calcifications in injured tendons, damaged cartilage or atheromatous vascular lesions, (II) crystal formation itself causes tissue injury and inflammation, for example in gouty arthritis, pulmonary silicosis or asbestosis, cholesterol crystals driving atherogenesis and in oxalate, cystine or urate nephropathy. Crystals trigger tissue inflammation via the NLRP3 inflammasome- and caspase-1-mediated secretion of IL-1β and IL-18 (refs 1, 2, 3, 4, 5). However, crystals also exert direct cytotoxic effects leading to necrotic rather than apoptotic cell death^6^,^7^. It is still unknown, whether crystal deposition causes necrosis in a passive-mechanical or in one of the recently identified modalities of regulated cell death^8^,^9^,^10^,^11^,^12^,^13^,^14^,^15^,^16^,^17^,^18^. We hypothesized that crystal-induced tissue injury involves a regulated form of cell death and that the identification of this pathway may reveal new targets for therapeutic intervention that limit crystal-related tissue injury and organ dysfunction. Our results show that various crystals uniformly induce necroptosis, that is, a receptor-interacting serine-threonine kinase 3- (RIPK3) and mixed lineage kinase domain-like (MLKL)-dependent form of primary necrosis *in vitro*, *in vivo* and in human disease. These data identify several molecular targets for pharmacological intervention to limit tissue injury in crystallopathies.

## Results

### Various crystals cause primary cellular necrosis

**Figure 1:**
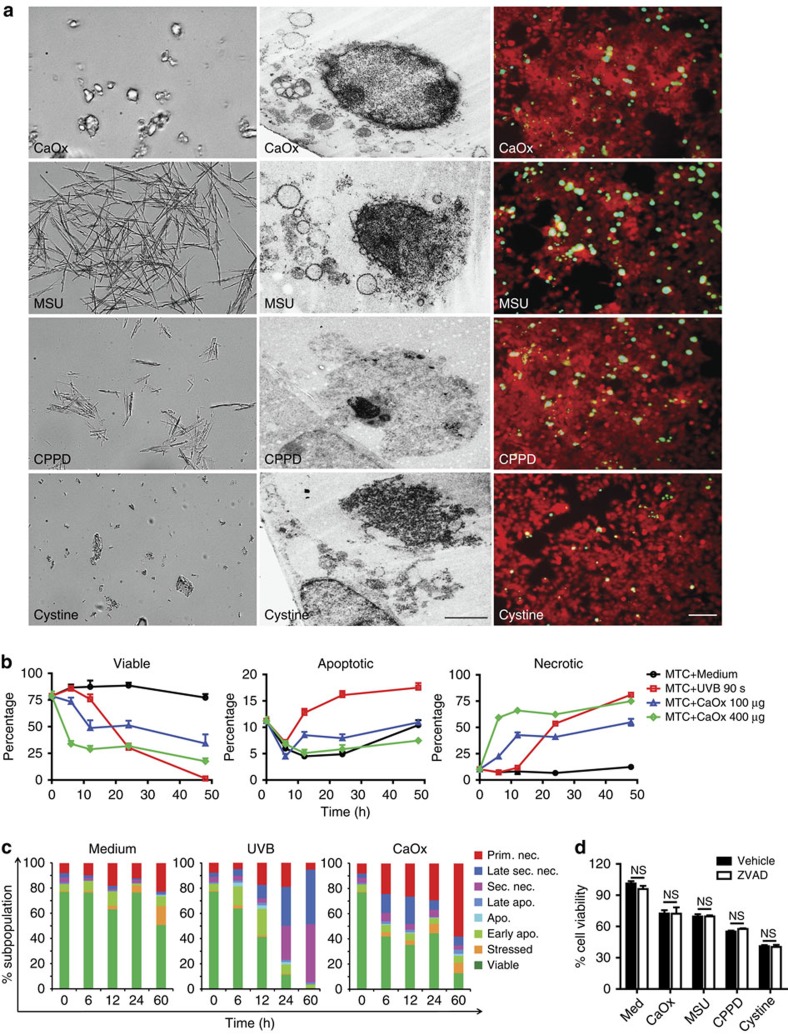
Crystals induce primary cell necrosis. (**a**): Various crystals were incubated with tubular epithelial cells as indicated. Images show the crystal shapes at a magnification of × 1,000 (left), TEM images shows that these crystals induce necrosis of tubular epithelial cells as indicated by ruptured plasma membranes (middle, × 2,000), scale bar, 2 μm. The images on the right show the same rhodamine-labelled monolayers (red) 24 h later. When Sytox green is added to the medium cells with permeable plasma membranes turn green indicating cell death (× 200), scale bar, 40 μm. (**b**) Flow cytometry was used to define the type and stage of tubular cell (MTC) death on CaOx crystal exposure or ultraviolet light type B for 90 s (s) over a period of 50 h as described in methods. Data for viable cells, apoptotic cells and necrotic cells are expressed as the percentage of viable cells±s.e.m. of all cells for each time point. (**c**): The graphs show a quantitative analysis of the same experiment displaying all different phenotypes of tubular epithelial cells. Flow cytometry cell death definitions are described in the methods section. (**d**) Mouse tubular epithelial cell viability on crystal exposure by 3-(4,5-dimethylthiazol-2-yl)-2,5-diphenyltetrazolium bromide assay with and without the pan-caspase inhibitor ZVAD–FMK–FMK. All data are mean±s.e.m. of at least three independent experiments. CaOx, MSU, CPPD, NS, not significant. *P<0.05 versus medium control, ***P<0.001 versus respective control. TEM, transmission electron microscopy.

What is the mode of crystal-induced cell death? To address this question we first studied the cytoxic effects of calcium oxalate (CaOx), monosodium urate (MSU), calcium pyrophosphate dihydrate (CPPD) and cystine crystals on kidney epithelial cells *in vitro*. Transmission electron microscopy images and rhodamine-sytox stains to identify living and dead cells, respectively, showed that kidney tubular epithelial cells die by primary necrosis after crystal exposure (Fig. 1a). In addition, we employed flow cytometry to better characterize the mode of CaOx-induced cell death. To this end sideward scatter, forward scatter, Hoechst 33342, annexin V, 1,1′-dioctadecyl-3,3,3′,3′-tetramethyl-indocarbocyanine perchlorate, and prodidium iodide were measured. We found that CaOx crystals induced primary necrosis without signs of apoptosis (Fig. 1b,c), evidenced by pan-caspase inhibition not affecting cytotoxicity of any of the aforementioned crystals (Fig. 1d). Also the kinetics of crystal cytotoxicity were caspase independent (Supplementary Fig. 1A) and absence of NLRP3 did not affect CaOx crystal-induced cytotoxicity (Supplementary Fig. 1B). This, being a caspase-independent mode of cell death, excluded NLRP3-caspase-1/11-dependent pyroptosis^10^,^11^. We conclude that crystal cytotoxicity does not involve apoptosis or pyroptosis but largely represents primary cellular necrosis.

### Cytotoxicity of crystals involves the necroptosis pathway

**Figure 2:**
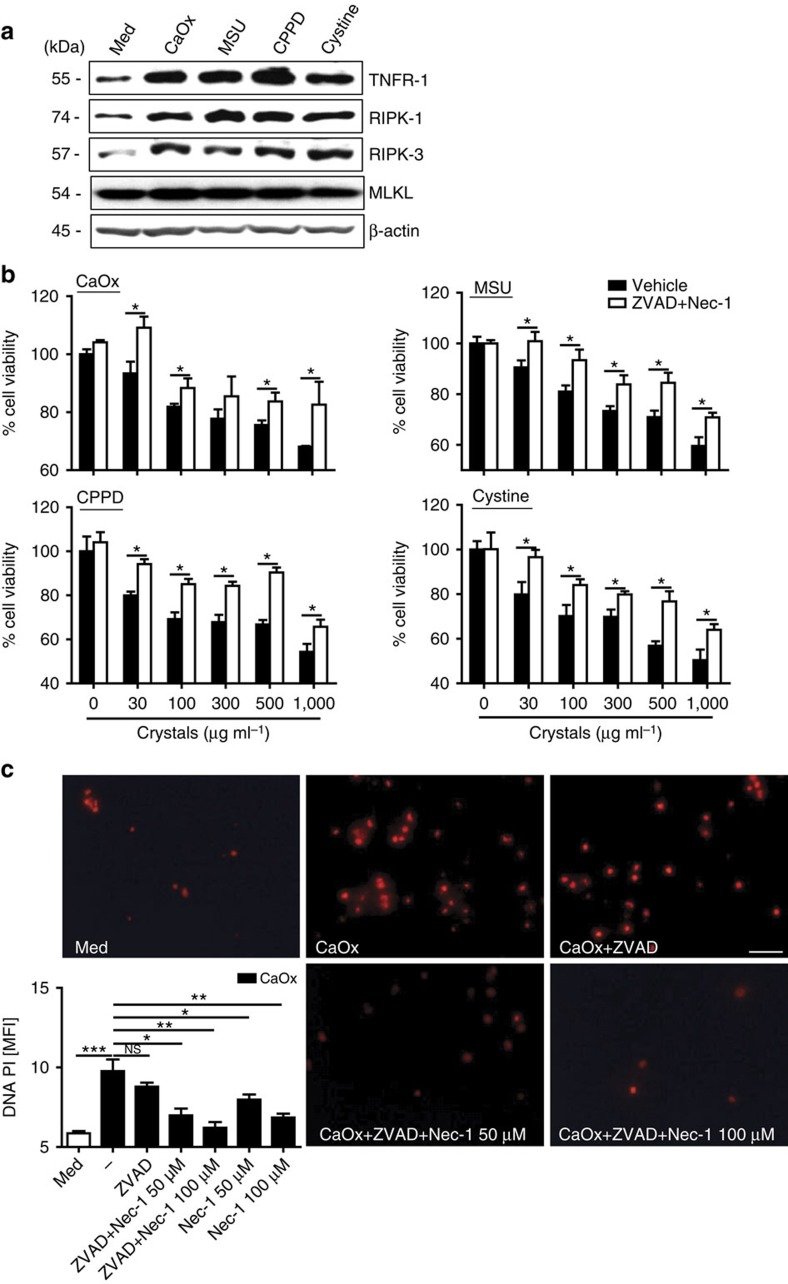
Crystal cytotoxicity involves the necroptosis pathway. (**a**): Protein expression of TNFR1, RIPK1 and RIPK3 was determined by western blot from total proteins isolated 18 h after stimulation of mouse tubular epithelial cells with crystals of CaOx (1,000 μg ml^−1^), MSU (500 μg ml^−1^), CPPD (500 μg ml^−1^) and cystine (500 μg ml^−1^). β-actin was used as loading control. (**b**) Mouse tubular epithelial cells were exposed to different concentrations of CaOx, MSU, CPPD or cystine crystals as indicated in the presence or absence of necrostatin (Nec)-1 (100 μM) together with the pan-caspase inhibitor ZVAD–FMK–FMK (10 μM). Cell viability was assessed by MTT assay 24 h later. Data are expressed as mean cell viability±s.e.m. of three independent experiments. Baseline viability is set as 100%. (**c**) In similar experiments necrotic tubular epithelial cells were identified via propidium iodide positivity and the results were expressed as mean fluorescent intensity on digital analysis of pictures taken from culture dishes. Representative images are shown at an original magnification of × 200, scale bar, 40 μm. Data were analysed using Student's *t*-test. *P<0.05, **P<0.01 and ***P<0.001 versus respective medium control. NS, not significant. MTT, 3-(4,5-dimethylthiazol-2-yl)-2,5-diphenyltetrazolium bromide.

**Figure 3:**
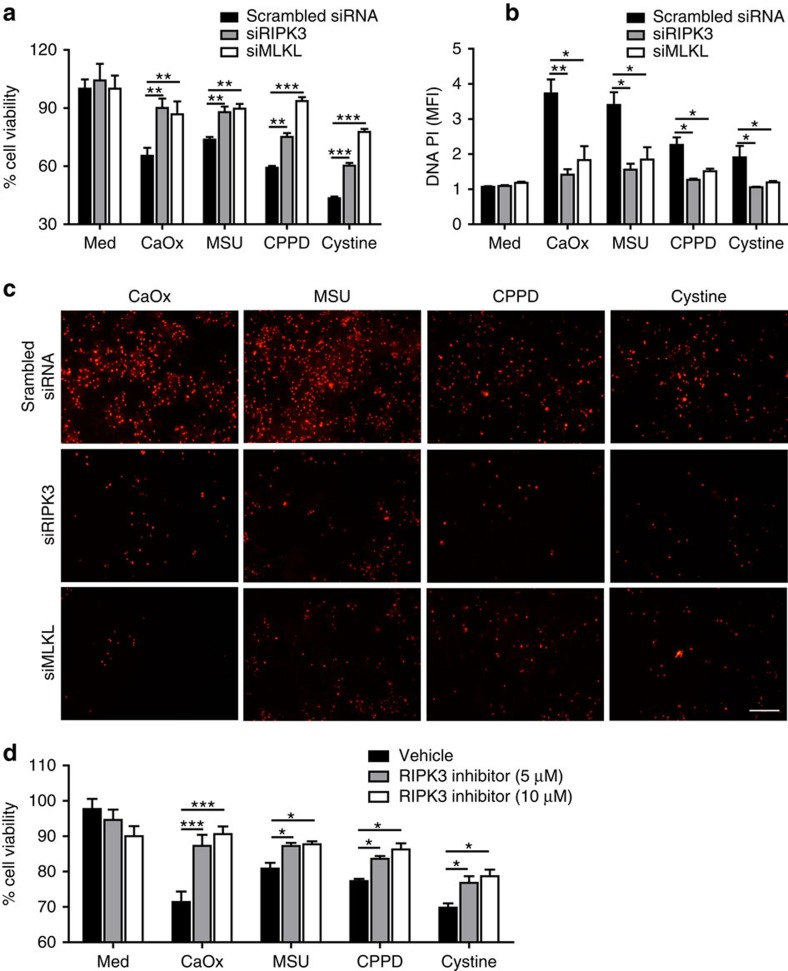
Suppression of RIPK3 and MLKL prevents crystal cytotoxicity. (**a**–**c**) Mouse tubular epithelial cells were transfected with specific small inhibitor (si) RNA for RIPK3 and MLKL or a control siRNA of scrambled sequence before being exposed to crystals of CaOx (1,000 μg ml^−1^), MSU (500 μg ml^−1^), CPPD (500 μg ml^−1^) and cystine (500 μg ml^−1^). Cell viability was assessed by MTT assay (a) and cell death was assessed quantifying PI positivity (b) and C shows representative images 24 h later. Original image magnification: × 200, scale bar, 100 μm. (**d**) Mouse tubular epithelial cells were pretreated with RIPK3 inhibitor dabrafenib before exposing to different type crystals. Cell viability was assessed by MTT assay 24 h later. Data are expressed as mean±s.e.m. of three independent experiments, and was analysed using Student's *t*-test. Baseline viability is set as 100%. *P<0.05, **P<0.01 and ***P<0.001 either versus control siRNA. MTT, 3-(4,5-dimethylthiazol-2-yl)-2,5-diphenyltetrazolium bromide; PI, prodidium iodide.

Having excluded the contribution of caspase-dependent forms of cell death, we focussed on necroptosis, a known regulated form of necrosis of non-immune cells^13^. We found the key proteins of the necroptosis pathway, that is, tumour-necrosis factor receptor-1 (TNFR1), RIPK1, RIPK3 and MLKL to be induced in tubular epithelial cells on exposure to various crystals (Fig. 2a). The RIPK1 stabilizer necrostatin-1 (ref. 19) partially (MSU, CPPD and cystine) or entirely (CaOx) prevented crystal-induced tubular epithelial cell death also in the absence of ZVAD–FMK (Fig. 2b and Supplementary Fig. 1C), suggesting a role for RIPK1 independent of caspases. When crystals were replaced by recombinant TNF-α as a stimulus, necrostatin-1 had the same effect. However, TNFR1 was not required for crystal-induced cell death *in vitro* (Supplementary Fig. 1B). Necrostatin-1 also suppressed crystal cytotoxicity in L929 cells, primary human synovial fibroblasts and HK-2 cells (Supplementary Fig. 2). The protective effect of necrostatin-1 on the cytotoxicity of crystals was confirmed by fluorescence imaging using the cell death marker prodidium iodide that enters cells only on the disruption of the plasma membrane (Fig. 2c). Both *Ripk3* or *Mlkl* deficiency or knockdown of either RIPK3 or MLKL with specific siRNA partially abrogated, whereas knockdown of caspase-8 somewhat enhanced crystal-induced cytotoxicity in tubular epithelial cells (Fig. 3a–c and Supplementary Fig. 3). Furthermore, pre-treatment with the putative inhibitor of RIPK3 dabrafenib also protected tubular epithelial cells from crystal-induced cytotoxicity in a dose-dependent manner (Fig. 3d). Together, these data imply that crystal-induced primary necrosis may involve RIPK3/MLKL-dependent necroptosis.

### *Ripk3* and *Mlkl* deficiency blocks crystalline necrosis *in vivo*

**Figure 4:**
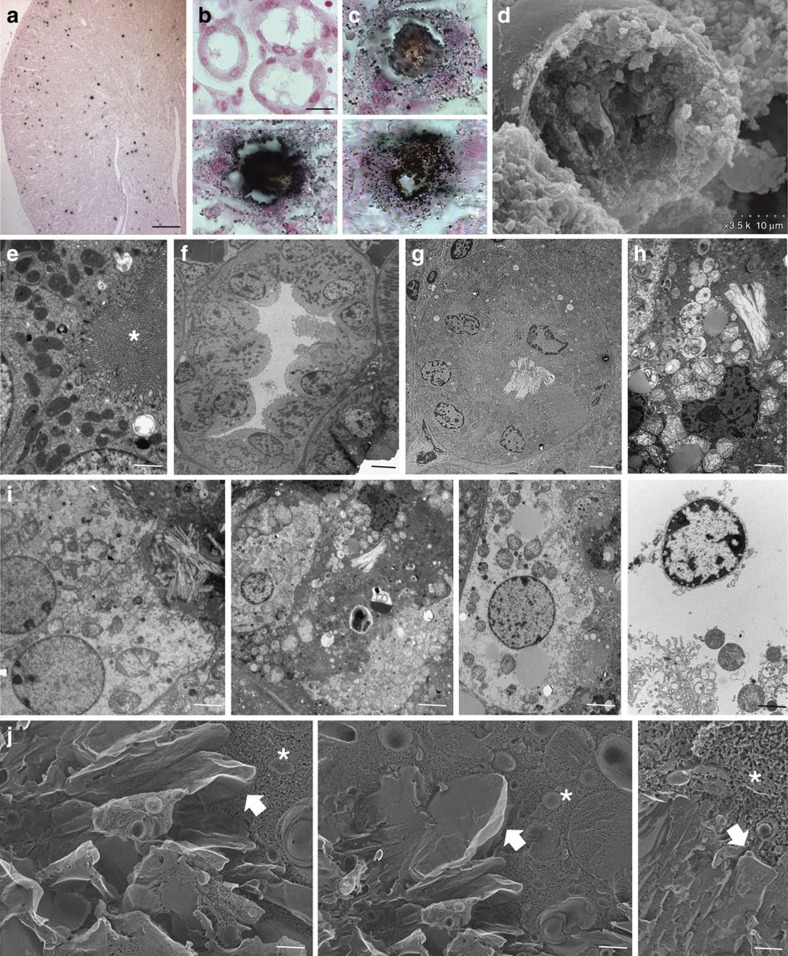
Acute oxalate nephropathy involves tubular epithelial cell necrosis. Oxalate exposure leads to diffuse intrarenal crystal formation as displayed by Pizzolato stain (**a**), scale bar, 1 mm. In contrast to healthy control mice (**b**) CaOx crystals form large stone-like plugs that obstruct the tubular lumen focally (**c**) smaller crystals attach to the luminal surface and also appear within the tubular cell cytoplasm. Scale bar, 20 μm (**d**) Scanning electron microscopy view of the lumen of CaOx exposed tubules confirms disruption of the proximal tubular brush boarder. Scale bar, 10 μm. Transmission electron microscopy shows intact proximal (**e**) and distal tubules (**f**) of control mice and luminal crystal plugs and intracellular crystals in mice exposed to CaOx (**g**). Scale bar, 5 μm. In many tubules crystal deposition is spatially associated with tubular epithelial cell necrosis, characterized by organelle swelling, cytoplasmic oedema and rupture of the plasma membrane with nuclear expulsion ((**h**) and all four panels of (**i**)). Scale bar, 2 μm. (**j**) Freeze fracture electron microscopy shows sheets of CaOx crystals (arrow head) in the cytoplasm (*) of tubular epithelial cells. Scale bar, 100 nm.

**Figure 5:**
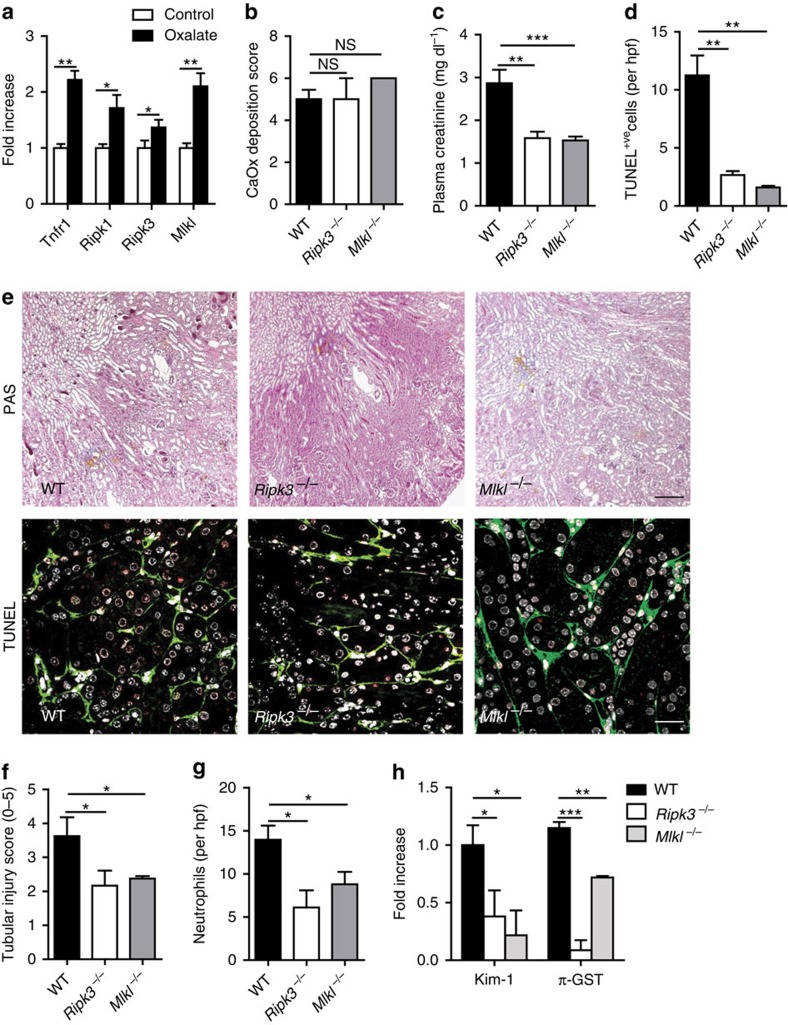
Oxalate nephropathy in C57BL/6 mice requires RIPK3 and MLKL. (**a**) Oxalate injection to C57BL/6 mice (*n*=5) induced intrarenal mRNA expression of TNFR1, RIPK1, RIPK3 and MLKL as determined by RT–PCR from total kidney isolates. (**b**) Oxalate feeding to wild-type mice as well as to *Ripk3*- and *Mlkl*-deficient mice of the same genetic background resulted in identical amounts of CaOx crystal deposition after 24 h as quantified by morphometry of Pizzolato-stained kidney sections. (**c**,**d**) Oxalate nephropathy in wild-type mice was associated with increased plasma creatinine (**c**) and TUNEL positive cells (**d**), which was both attenuated in the gene deficient mouse strains. (**e**) PAS staining illustrated tubular necrosis at the corticomedullary junction in wild-type mice. Original image magnification: × 100. TUNEL staining identified dying cells (red), with counterstaining for laminin (green) and cell nuclei (DAPI, white). Original image magnification: × 200. Scale bar, 0.5 mm (upper panel); 20 μm (lower panel). (**f**) Tubular injury was quantified by semiquantitative scoring of PAS-stained section as described in methods. (**g**) Neutrophils were identified by immunostaining and counted per high power field. (**h**) RT–PCR from total kidney isolates quantified intrarenal mRNA expression of the tubular injury markers Kim-1 and *π*-GST. Data are means±s.e.m. from five mice in each group. Data were analysed using one-way ANOVA with *post hoc* Bonferroni's correction. *P<0.05, ***P<0.001 versus wild-type mice. ANOVA, analysis of variance; PAS, periodic acid-Schiff; RT–PCR, reverse transcription PCR.

Crystal nephropathy (CN) is an example of crystal-induced tissue injury and organ failure^20^,^21^. Mice exposure to oxalate induces oxalate nephropathy, a model that mimics CN in humans, including an increase of serum creatinine and blood urea nitrogen (BUN)^5^,^20^,^22^. Acute oxalate exposure leads to CaOx crystal deposition in the mouse kidney. At the structural level CaOx crystal plugs forms within the proximal and distal tubules (Fig. 4a–d). CaOx crystals are also found within the interstitium (Fig. 4c). Transmission electron microscopy detected luminal crystal plugs and smaller sized crystals within tubular epithelial cells (Fig. 4e–g). Injured epithelial cells often exhibited ultrastructural signs of necrosis, such as loss of electon density and balloning of the mitochondria, nuclear chromatin irregularities, outer membrane disruption, and cytoplasm and organelle expulsion (Fig. 4h). Quick-freeze deep-etch electron microscopy illustrates crystal deposition involving cytoplasmic organelles on rupture of plasma membranes (Fig. 4i). Thus, crystal formation within the kidney is associated with necrosis of tubular epithelial cells in a temporal and spatial manner. The phenotype is associated with the induction of renal expression of TNFR1, RIPK1, RIPK3 and MLKL messenger RNA (mRNA) (Fig. 5a). We validated the *in vivo* contribution of this pathway by inducing oxalate nephropathy in wild type and *Ripk3*- and *Mlkl*-deficient mice. Oxalate exposure induced identical CaOx crystal deposits in both mouse strains (Fig. 5b), but all functional and structural parameters of acute CN were significantly reduced in *Ripk3*- or *Mlkl*-deficient mice (Fig. 5c–h). These include serum creatinine levels, markers of tubule necrosis and neutrophil recruitment. These results show that the necroptosis-related proteins RIPK3 and MLKL mediate oxalate crystal-induced cell necrosis *in vivo*.

### RIPK1 does not mediate neutrophil recruitments

**Figure 6:**
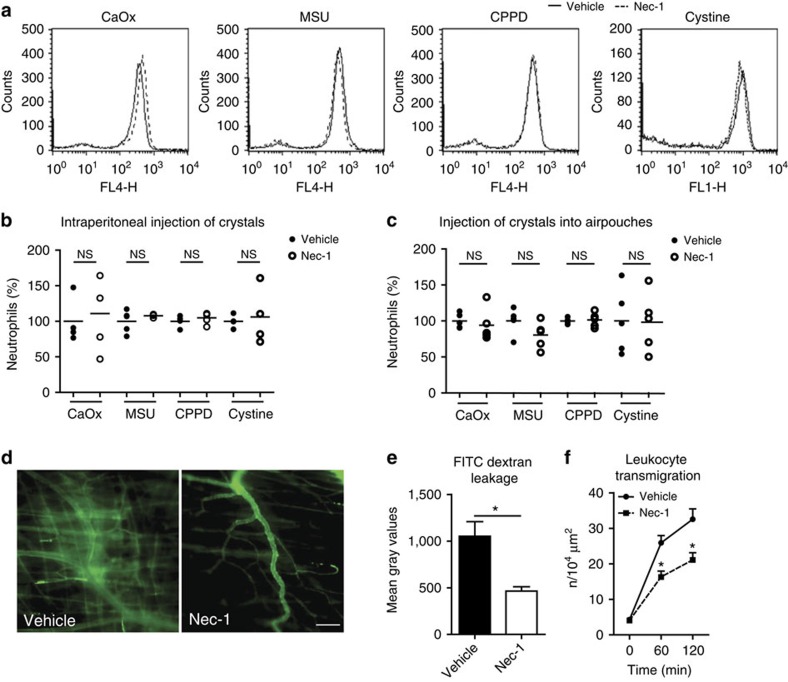
Necrostatin-1 and neutrophil recruitment. (**a**,**b**): i.p. injection of CaOx, MSU, CPPD or cystine crystals into C57BL/6 mice (*n*=4 in each group) induces neutrophil recruitment to the peritoneal cavity as determined by flow cytometry of peritoneal lavage fluids. This process is not affected by concomitant necrostatin-1 treatment. A shows representative flow cytometry data for each crystal presented as mean fluorescence intensity for the neutrophil marker. (**b**) Shows data of each mouse with the mean of wild-type mice set as 100%. NS, not significant. (**c**) Similar experiments using the air pouch model of neutrophil recruitment gave identical results (*n*=5 in each group) and are expressed in the same manner. (**d**–**f**) *In vivo* microscopy studies of the postischemic musculus cremaster were performed as a second model of injury-associated and ROS-dependent neutrophil recruitment in C57BL/6 mice. Necrostatin-1 significantly reduced the microvascular leakage of FITC-labelled dextran particles as illustrated by representative images in D at an original magnification of × 400, scale bar, 100 μm and quantitatively in (**e**). In the same experiment leukocyte transmigration from the microvasculature was quantified by counting from video recordings taken at baseline and at 60 and 120 min. Data are means±s.e.m. from seven mice in each group. Data were analysed using one-way ANOVA with *post hoc* Bonferroni's correction. *P<0.05, **P<0.01, ***P<0.001 versus vehicle control. ANOVA, analysis of variance.

We performed several *in vitro* and *in vivo* experiments to test the possibility that tubule protection in *Ripk3*- and *Mlkl*-deficient mice is a secondary effect possibly due to a role of RIPK1–RIPK3–MLKL signalling in neutrophil recruitment. Intraperitoneal (i.p.) injection of various types of crystals induced neutrophil recruitment into the peritoneal cavity as a marker of crystal-induced peritonitis, which was not affected by necrostatin-1 (Fig. 6a,b). Injection of crystals into air pouches at the back of mice with and without systemic necrostatin-1 treatment gave identical results (Fig. 6c and Supplementary Fig. 4). Importantly, we assured the plasma activity of the same charge of necrostatin-1 *in vivo* in a similar time frame by testing microvascular permeability and leucocyte extravasation during postischemic cremaster muscle injury (Fig. 6d–f). We conclude that the necrostatin-1-mediated effects on tissue injury do not involve a direct effect on neutrophil recruitment.

### TNFR1 triggers tubular cell necroptosis *in vivo*

**Figure 7:**
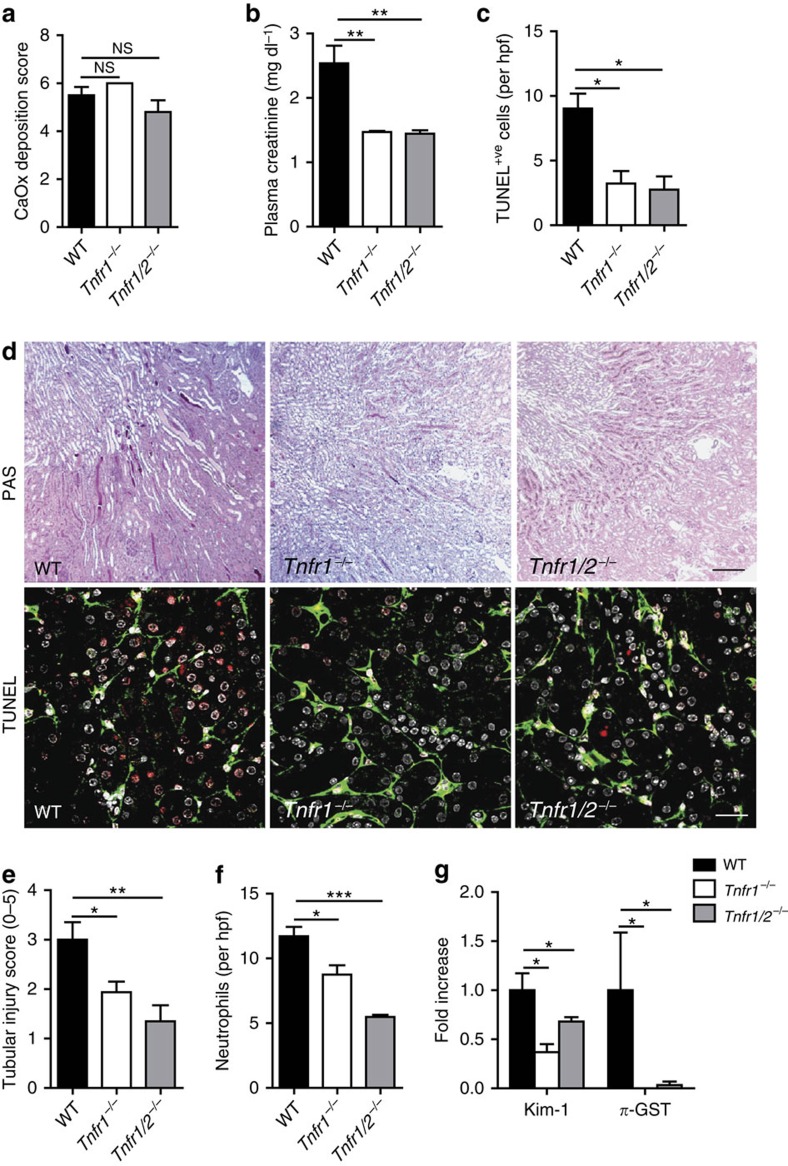
Oxalate nephropathy in C57BL/6 mice requires TNFR1. (**a**) Oxalate injection to wild-type mice as well as to *Tnfr1*- and *Tnfr1/2*-deficient mice of the same genetic background (*n*=5 in each group) resulted in identical amounts of CaOx crystal deposition after 24 h as quantified by morphometry of Pizzolato-stained kidney sections. (**b**,**c**) Oxalate nephropathy in wild-type mice was associated with increased plasma creatinine levels (**b**) and TUNEL positive cells (**c**), which were attenuated to similar extend in both gene deficient mouse strains. (**d**) PAS staining illustrated tubular necrosis at the corticomedullary junction in wild-type mice. Original image magnification: × 100. TUNEL staining identified dying cells (red), with counterstaining for laminin (green) and cell nuclei (DAPI, white). Original image magnification: × 200. Scale bar, 0.5 mm (upper panel), 20 μm (lower panel). (**e**) Tubular injury was quantified by semiquantitative scoring of PAS-stained section as described in methods. (**f**): Neutrophils were identified by immunostaining and counted per high power field. (**g**) RT-PCR from total kidney isolates quantified intrarenal mRNA expression of the tubular injury markers Kim-1 and *π*-GST. Data are means±s.e.m. from five mice in each group. Data were analysed using one-way ANOVA with *post hoc* Bonferroni's correction. *P<0.05, ***P<0.001 versus wild-type mice. ANOVA, analysis of variance; RT–PCR, reverse transcription PCR.

Crystals did not require TNFR1 to induce tubular epithelial cell necroptosis *in vitro*, necrosis-driven renal inflammation (‘necroinflammation') may induce additional TNF-α-triggered necroptosis *in vivo*, for example, via surface TNFR1 on tubular epithelial cells. Renal mRNA and protein levels of TNF-α and TNFR1 were induced in CaOx nephropathy at 24 h (Supplementary Fig. 5A–C). A careful analysis of early time points of oxalate nephropathy revealed that kidney injury occurred several hours before intrarenal TNF expression (Supplementary Fig. 6). Flow cytometry revealed that TNF-α was not only expressed by intrarenal mononuclear phagocytes but also by non-immune cells (Supplementary Fig. 5D). Immunostaining localized TNF-α expression to tubules but also interstitial cells, while TNFR1 and -2 were mostly induced in tubular epithelial cells (Supplementary Fig. 5E). To test for a possible functional contribution of TNFR signalling we induced CN in *Tnfr1*-deficient and *Tnfr1/2* double-deficient mice. Oxalate exposure resulted in identical CaOx crystal deposits in all mouse strains, however, all functional and structural parameters of acute CN were significantly reduced in *Tnfr1*-deficient mice (Fig. 7). These include serum creatinine levels, BUN, tubule necrosis and neutrophil recruitment. Concomitant deficiency in TNFR2 had no additive effects on this phenotype, suggesting that TNFR1 mainly accounts for this effect. Thus, although direct crystal-induced necroptosis does not require TNFR1, *in vivo*, crystal-induced necroinflammation involves additional TNFR1 signalling-related cell death^23^, which contributes to CN.

### Novel therapeutic targets to abrogate crystal nephropathy *in vivo*

**Figure 8:**
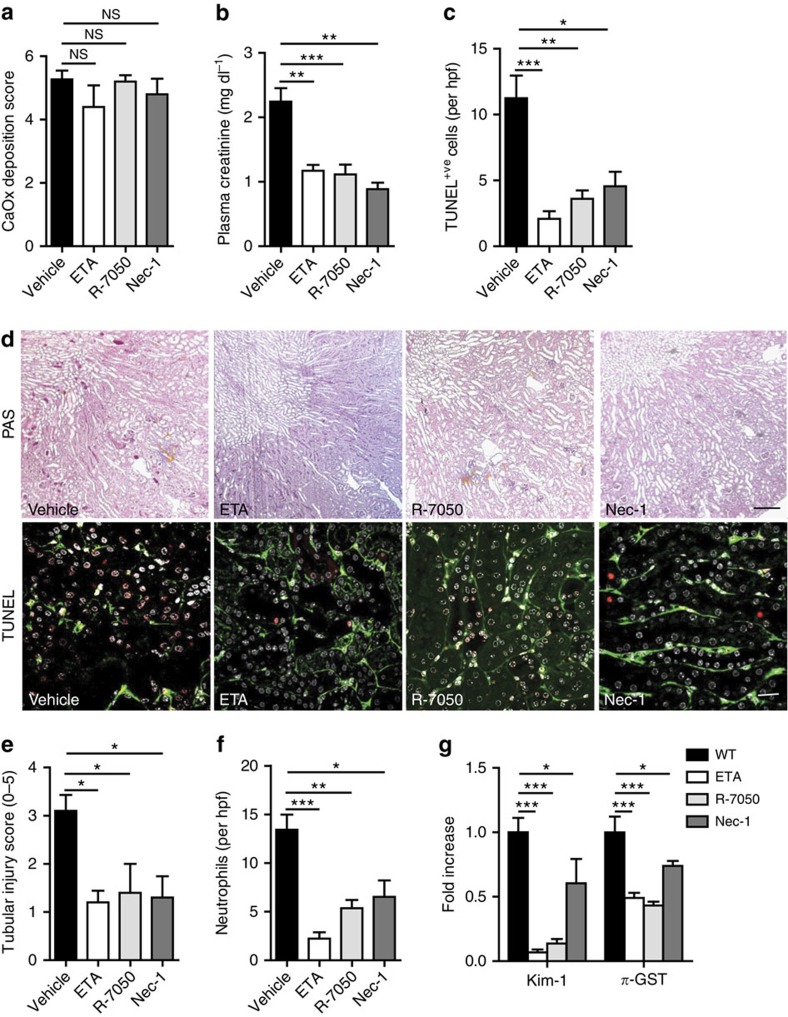
Etanercept, R-7050, and necrostatin-1 abrogate crystal nephropathy. (**a**–**c**): Oxalate injection to wild-type mice treated either with vehicle, the TNF-α blocker etanercept, the TNFR blocker R-7050 or necrostatin (Nec)-1 (*n*=5 in each group) resulted in identical amounts of CaOx crystal deposition after 24 h as quantified by morphometry of Pizzolato-stained kidney sections (**a**) but significantly reduced the levels of plasma creatinine (**b**) and the number of TUNEL positive cells in kidney sections (**c**). (**d**) PAS staining illustrated tubular necrosis at the corticomedullary junction in wild-type mice. Original image magnification: × 100. TUNEL staining identified dying cells (red), with counterstaining for laminin (green) and cell nuclei (DAPI, white). Original image magnification: × 200. Scale bar, 0.5 mm (upper panel), 20 μm (lower panel). (**e**) Tubular injury was quantified by semiquantitative scoring of PAS-stained section as described in methods. (**f**) Neutrophils were identified by immunostaining and counted per high power field. (**g**) RT–PCR from total kidney isolates quantified intrarenal mRNA expression of the tubular injury markers Kim-1 and *π*-GST. Data are means±s.e.m. from five mice in each group. Data were analysed using one-way ANOVA with *post hoc* Bonferroni's correction. *P<0.05, ***P<0.001 versus vehicle-treated mice. ANOVA, analysis of variance; RT–PCR, reverse transcription.

If TNFR1/RIPK1/RIPK3/MLKL-mediated necroptosis drives tubule necrosis in CN then inhibition of either TNF-α, TNFR signalling or RIPK1 might be valuable therapeutic targets. To test this hypothesis we treated mice with the TNF-α blocker etanercept, the TNFR internalization inhibitor R-7050, or with necrostatin-1. Oxalate exposure resulted in equal amounts of CaOx crystal deposits in all groups of mice but etanercept, R-7050 or necrostatin-1 significantly reduced all functional and structural aspects of CN, that is, serum creatinine levels, tubule necrosis, neutrophil influx and the mRNA expression of kidney injury markers (Fig. 8). Thus, crystal-induced TNF-α release and the subsequent TNFR1-triggered activation of RIPK1–RIPK3–MLKL-executed tubular cell necroptosis represent therapeutic targets in CN. Using etanercept, R-7050 or necrostatin-1 may have therapeutic potential.

### Phosphorylated MLKL in human acute oxalate nephropathy

**Figure 9:**
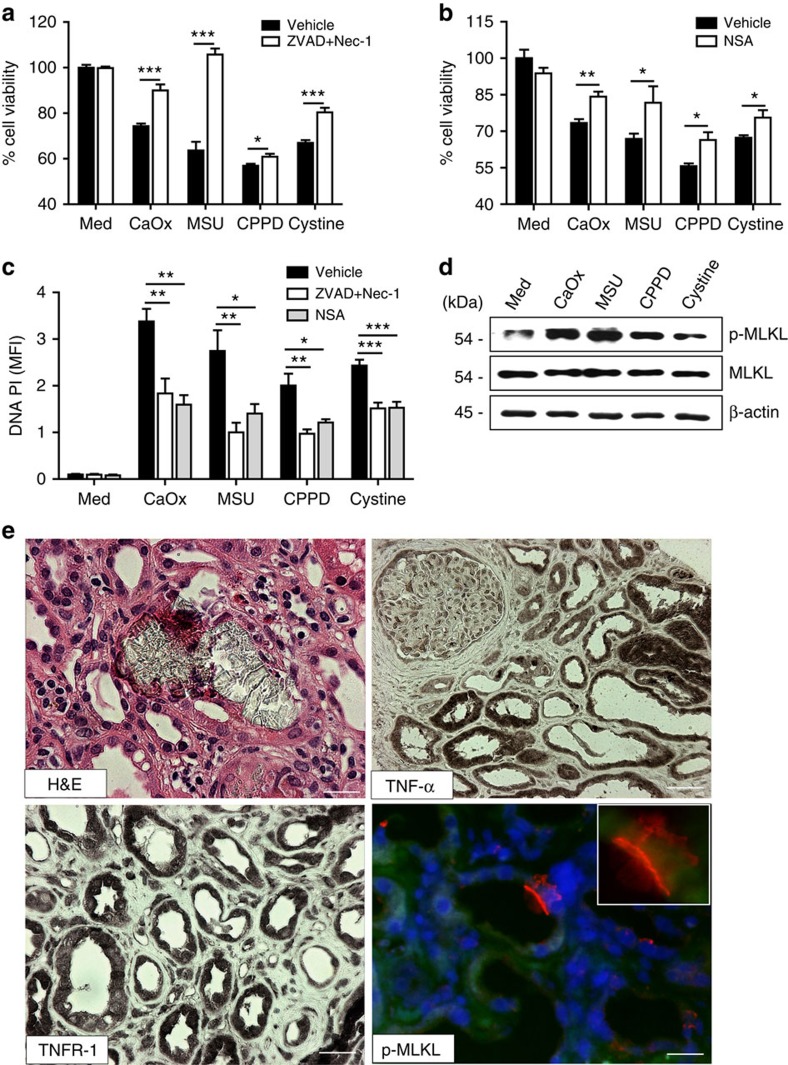
Necroptosis is involved in human acute oxalate nephropathy. (**a**–**c**) Primary renal human progenitor cells were pretreated with either ZVAD–FMK (10 μM) and Nec-1 (100 μM) or NSA (1 μM) before being exposed to CaOx (1000 μg ml^−1^), MSU (500 μg ml^−1^), CPPD (500 μg ml^−1^) and cystine (500 μg ml^−1^). Cell viability was assessed by MTT assay (**a** and **b**) and cell death was assessed quantifying PI positivity (**c**) 24 h later. Data are expressed as mean±s.e.m. of three independent experiments. Baseline viability is set as 100%. Data were analysed using Student's *t*-test. *P<0.05, **P<0.01, ***P<0.001 either versus vehicle control. (**d**) Primary renal human progenitor cells were stimulated with different crystals as indicated above for 18 h. The expression of Phospho-mlkl and total mlkl was detected by western blot with β-actin as a loading control. (**e**) Selective cases of human oxalate crystal-related acute kidney injury were stained with PAS and for TNF-α, TNFR1 and phosphorylated MLKL. Representative images are shown at an original magnification of 1:400. Scale bar, 40 μm. MTT, 3-(4,5-dimethylthiazol-2-yl)-2,5-diphenyltetrazolium bromide; PAS, periodic acid-Schiff; PI, prodidium iodide.

To test validate our mouse data in human disease we first analysed crystal cytotoxicity in human renal progenitor cells, that are considered to contribute to the turnover of tubular epithelial cells and their regeneration upon injury^24^. For all the crystals tested the results were identical to the other cell types examined before (Fig. 9a–d and Supplementary Fig. 7). Of 64,000 renal biopsies, 1,119 cases (1.7%) with documented CaOx crystals were identified by light microscopy and birefringence under polarized light. In all, 412 of these 1,119 cases (37 or 0.6% of total) biopsy cases showed CaOx crystals in association with acute tubular injury, which represented 10% of all 4,125 biopsy cases with acute kidney injury as the leading clinical diagnosis. Immunostaining revealed diffuse tubular positivity for TNF-α, TNFR1 and total MLKL, while phosphorylated MLKL was detected only in single cells (Fig. 9e). We conclude that crystal-induced cytotoxicities of human and murine kidney cells are comparable and that human tubular epithelial cells also activate MLKL in the context of oxalate crystal-induced acute kidney injury.

## Discussion

Various crystals trigger cell death and inflammation. While inflammation was traditionally thought to be secondary to tissue injury, the discovery of crystals being agonists of the NLRP3 inflammasome raises the question whether crystal cytotoxicity is a consequence of NLRP3 activation or an inflammasome-independent event with distinct signalling pathways^1^. Crystals are NLRP3 agonists, which could imply triggering also NLRP3-caspase-1/11-mediated pyroptosis, a mode of regulated cell death occurring in infected macrophages^10^,^11^,^12^. We excluded this option as *Nlrp3*-deficiency or caspase blockade did not affect crystal cytotoxicity. Caspase blockade virtually also excluded apoptosis as another form of regulated cell death, which was consistent with our flow cytometry results and the results of a previously published report^6^. In contrast, we found that the cytotoxicity of crystals involves necroptosis, an injury-driven form of regulated cell death driven by the phosphorylation of the pseudokinase MLKL^13^,^14^,^25^,^26^,^27^. Knockdown of RIPK3 and MLKL almost entirely abrogated crystal cytotoxicity *in vitro*, suggesting strongly that necroptosis is the predominant form of crystal-induced cell death^13^,^14^,^28^. As further lines of evidence, lack of *Ripk3* and *Mlkl* also almost entirely abrogated oxalate nephropathy *in vivo*. These data further support the concept that cell necrosis is the initial event of crystal-induced necroinflammation and that blocking necroptosis during the early stage of tissue injury is sufficient to prevent subsequent auto-amplification loop of inflammation, immune-mediated pathology and organ failure^29^. However, RIPK3 is also involved in NLRP3 inflammasome activation^30^, which is an important trigger of tissue inflammation in oxalate nephropathy^5^. Of note NLRP3 and ASC deficiency both lacked oxalate-induced kidney injury, and was exclusively mediated by intrarenal dendritic cells, which excludes any inflammasome signalling in non-immune kidney cells. Therefore, the *in vivo* phenotype of the RIPK3 knockout may also involve impaired NLRP3/ASC signalling in intrarenal dendritic cells.

Providing evidence that necroptosis is also involved in human disease is difficult because the different forms of regulated necrosis cannot be distinguished by ultrastructural morphological criteria. To resolve this issue we used immunostaining for phosphorylated MLKL, which suggests MLKL-dependent regulated necrosis also in human oxalate nephropathy.

Necrostatin-1 keeps RIPK1 in an anti-necroptotic conformation and protected from crystal-induced cytotoxicity. As RIPK1 interacts with the intracellular domain of TNFR1 via its death domain^31^ and TNFR1-mediated necroptosis is considered a prototype of regulated necrosis^8^, it is tempting to speculate that RIPK1 loses its protective function against crystal-induced organ failure presumably by post-translational events, such as deubiquitination on TNFR1 ligation. Consistently, lack of TNFR1 or treatment with etanercept abrogated CN *in vivo*, but not crystal-induced cytotoxicity *in vitro*. This should relate to the lack of direct interaction between crystals and TNFR1 and the absence of TNF-α in the *in vitro* system (Supplementary Fig. 8). *In vivo*, however, TNF-α is secreted by infiltrating immune cells, which triggers necroptosis in additional cell populations secondary to the initial direct activation by crystals, thus contributing to the auto-amplification loop of necroinflammation^29^. The selective expression of TNF-α and TNFR1 in tubules of human oxalate nephropathy implies a similar role also in human disease.

How exactly crystals trigger RIPK1 activation remains uncertain. MSU crystals can induce Syk signalling in dendritic cells via biding to lipid rafts^32^. MSU crystals also affect signalling by binding to the surface receptor Clec12A but this interaction does not apply to other crystals^33^. Crystals activate the NLRP3 inflammasome via lysosomal leakage of cathepsins into the cytosol^34^. Cathepsin B was reported to inhibit necroptosis by cleaving RIPK1 (ref. 35). This could also be an avenue of crystal-induced necroptosis.

**Figure 10:**
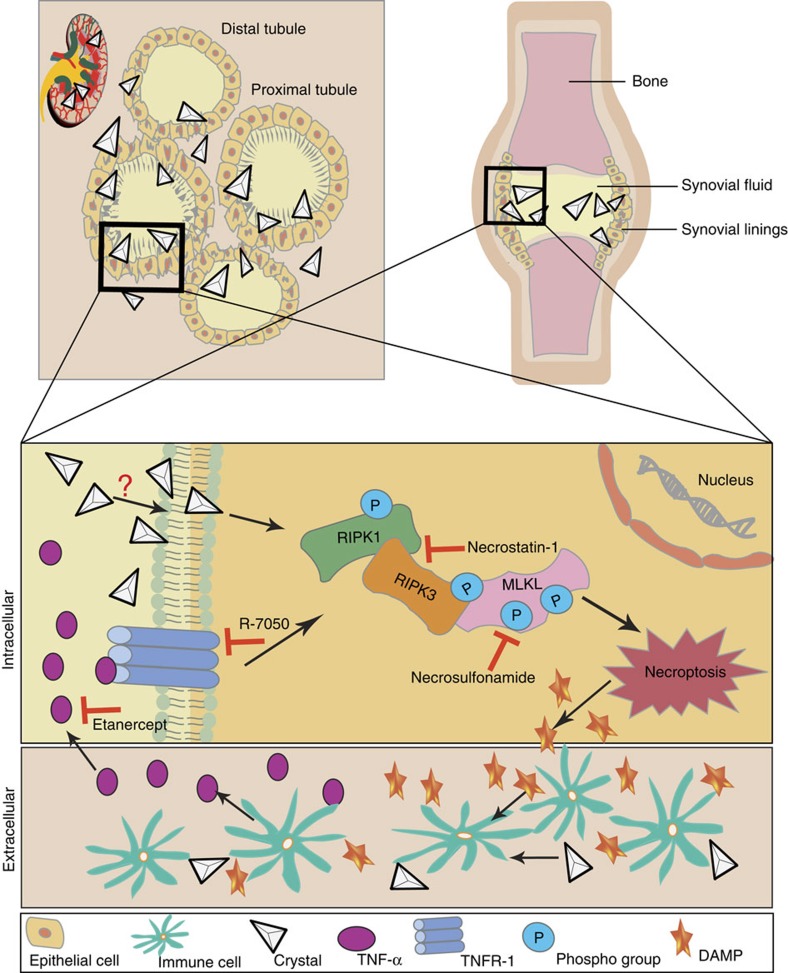
Schematic illustration of crystal-induced necroptosis and inflammation (necroinflammation). During crystallophathies, crystals are formed and deposited inside the organ, for example, kidney stone disease or joint, for example, gouty arthritis. Upon crystallization, crystals are phagocytized by parenchymal cells where they activate the RIPK1, RIPK3 and MLKL pathway of necroptosis, a prototype form of regulated necrosis, by inducing a series of phosphorylation events. Cellular necroptosis lead to release of DAMPs, which activate immune cells surrounding the parenchymal cells. Activated immune cells release pro-inflammatory cytokines, for example, IL-1β, IL-6 and TNF-α and so on. Pro-inflammatory cytokines like TNF-α can also activate the RIPK1, RIPK3 and MLKL pathway of necroptosis via TNFR1. The auto-amplification loop between cell death and inflammation, called necroinflammation, leads to aggravation of tissue injury, and if remain uncontrolled then to organ failure. In the current settings, the loop of crystal-induced necroinflammation can be blocked by using a soluble TNFR1-hIgG1 fusion protein etanercept to inhibit TNF-α, TNFR inhibitor R-7050, RIPK1 inhibitor necrostatin-1 or MLKL inhibitor necrosulfonamide. RIPK, receptor-interacting protein kinase; MLKL, mixed lineage kinase domain like; DAMPs, danger associated molecular patterns; IL, interleukin; TNF, tumour-necrosis factor; TNFR, tumour-necrosis factor receptor.

In conclusion, crystal cytotoxicity involves necroptosis as a form of regulated cell death *in vitro* and *in vivo*. *In vivo*, also secondary TNF-driven necroptosis adds in (Fig. 10). Consequently, the therapeutic blockade of this pathway, for example, with soluble TNFR2-IgG fusion protein or the RIPK1 stabilizer necrostatin-1, can prevent crystal-induced tissue necrosis and organ dysfunction. These findings imply TNFR1, RIPK1, RIPK3 and MLKL being potential therapeutic targets to limit tissue injury in crystallopathies.

## Methods

### Animal studies

C57BL/6N mice were procured from Charles River Laboratories (Sulzfeld, Germany). *Tnfr1*−/− and *Tnfr*2−/− mice were originally obtained from the Jackson Laboratories (Bar Harbour, ME) and bred under specific pathogen-free conditions. *Tnfr1*/2 double-deficient mice (*Tnfr1,2*−/−) were generated by cross-breeding *Tnfr1*−/− and *Tnfr2*−/− mice. *Ripk3*−/− mice were kindly provided by V. Dixit, Genentech, USA and *Mlkl*−/− mice were kindly provided by J. Murphy, WEHI, Australia. Mice were housed in groups of five under specific pathogen-free conditions with unlimited access to food and water. Six- to eight-week-old male mice received a single intraperitoneal (i.p.) injection of 100 mg kg^−1^ of sodium oxalate (Santa Cruz Biotechnology, USA) and 3% sodium oxalate in drinking water and kidneys were harvested after 24 h. As a therapeutic strategy, mice received a single dose of either etanercept (10 mg kg^−1^ i.p., Pfizer, Germany) or necrostatin-1 (1.65 mg kg^−1^ i.p., Millipore, Germany) or R-7050 (18 mg kg^−1^ i.p., Santa Cruz Biotechnology, USA) before sodium oxalate injections. Blood, urine and kidneys were collected at sacrifice by cervical dislocation. Kidneys were kept at −80 °C for protein isolation and in RNA later solution at −20 °C for RNA isolation. One part of the kidney was also kept in formalin to be embedded in paraffin for histological analysis. Samples for electron microscopy were fixed in glutaraldehyde. Sample sizes were determined by G*Power (Germany). All kidney disease experimental procedures were approved by the Regierung von Oberbayern, München, Germany.

For peritonitis studies, peritonitis was induced by injection of 1 mg of crystals in 0.5 ml sterile PBS. Mice received a single injection of either Nec-1 (1.65 mg kg^−1^) or vehicle half an hour before crystals injections. After 6 h, mice were killed and peritoneal cavities were washed with 5 ml of PBS. The lavage fluids were analysed for polymorphonuclear neutrophil recruitment by fluorescence-activated cell sorting using the neutrophil marker Ly-6G (BD Biosciences, Germany).

For air pouch studies, murine air pouches were generated using standard protocols in groups of 6-weeks-old Balb/c mice (*n*=5). In brief, we injected 3 ml of sterile air subcutaneously into the back of anaesthetized mice to form an air pouch. An additional 2 ml of sterile air was injected into pre-existent pouch 3 days after the first injection^7^. Another 2 days later, we injected 2.5 mg crystals in PBS, 2.5 mg crystals plus 1.65 mg kg^−1^ necrostatin-1 into the air pouches. Crystals of CaOx, MSU, CPPD and cystine were used in each independent experiment. After 24 h the pouch fluid was collected, and neutrophils in the air lavage were analysed by fluorescence-activated cell sorting. The studies were conducted according to guidelines determined by the Law of the Ministry of Healthcare of Ukraine, No. 281 from 1 November 2011 for the care and use of laboratory animals and were approved by the Ethics Council of the Danylo Halytsky Lviv National Medical University.

For mouse cremaster muscle IR injury assay, cremaster muscle was prepared for experiment as originally described by Baez with minor modifications^36^,^37^. Briefly, mice were anesthetized using intraperitoneal (i.p.) injection of ketamine/xylazine mixture. The left femoral artery was cannulated for administration of microspheres and drugs. The right cremaster muscle was exposed through a ventral incision of the scrotum. Epididymis and testicle were detached from the cremaster muscle and placed into the abdominal cavity. Throughout experimental procedure the muscle was superfused with warm-buffered saline. Olympus BX 50 upright microscope (Olympus Microscopy, Germany) equipped for stroboscopic fluorescence epi-illumination microscopy was used for *in vivo* microsopy. For measurement of centerline blood flow velocity, green fluorescent microspheres (0.96 μm diameter, Molecular Probes, Leiden, the Netherlands) were injected via the femoral artery catheter, and their passage through the vessels of interest was recorded using the fluorescein isothiocyanate (FITC) filter cube under appropriate stroboscopic illumination (exposure, 1 ms; cycle time, 10 ms; *l*=488 nm), integrating video images for sufficient time (>80 ms) to allow for the recording of several images of the same bead on one frame. Beads that were flowing freely along the centerline of the vessels were used to determine blood flow velocity. For offline analysis of parameters describing the sequential steps of leucocyte extravasation, we used the Cap-Image image analysis software (Dr Zeintl, Germany). Animals were treated with necrostatin-1 (1.65 mg kg^−1^ i.p.) or vehicle 30 min before the experiment. For the analysis of postischemic leucocyte responses, three postcapillary vessel segments in a central area of the spread out cremaster muscle were randomly chosen. Ischaemia was induced by clamping all supplying vessels at the base of the cremaster muscle using a vascular clamp (Martin, Germany). After 30 min of ischaemia, the vascular clamp was removed and reperfusion was restored for 160 min. Measurements were repeated at 60 and 120 min after onset of reperfusion. Subsequently, FITC dextran was infused intra-arterial for the analysis of microvascular permeability. Analysis of microvascular permeability was performed as follows^38^. The macromolecule FITC dextran (5 mg in 0.1 ml saline, Mr 150,000 Da, Sigma Aldrich) was infused intra-arterial after determination of centerline blood flow velocity. Mean grey values of fluorescence intensity were measured by digital image analysis (TILLvisION 4.0, TILL Photonics) in six randomly selected regions of interest (50 × 50 μm^2^), localized 50 μm distant from the postcapillary venule under investigation. The average of mean grey values was calculated.

### Assessment of renal injury

Mice kidney sections of 2 μm were stained with periodic acid-Schiff reagent. Tubular injury was scored by assessing the percentage of necrotic tubules and presence of tubular casts. Ly6B.2+ neutrophils were identified by immunostaining (1:200; Serotec, UK, #MCA771GA). Pizzolato's staining visualized CaOx crystals and crystal deposit formation in the kidney was evaluated as in refs 5, 39: no deposits=0 point; crystals in papillary tip=1 point; crystals in cortical medullary junction=2 points; crystals in cortex=3 points. When crystals were observed in multiple areas, the points were combined. The expression of TNF-α (1:200, R&D, Germany, #AF410NA) and TNFR1 (1:800, Abcam, UK, #ab19139) was analysed by immunostaining. Plasma creatinine and BUN were measured using Urea FS kit (DiaSys Diagnostic Systems, Germany) according to the manufacturer's protocol. Neutrophil infiltrates were counted in 15 high power fields per section. A cell death detection (TUNEL) kit (Roche, Germany) was used to quantify dead cells. A retrospective screening for CaOx deposits in 64,000 medical and transplant kidney biopsies renal biopsies in the Nephropath data base was performed (IRB number: 05-4397-0). An informed consent was obtained from all subjects. Paraffin sections from human native kidney biopsies were stained with 1:100 rabbit anti p-MLKL (Abcam, USA) and anti-rabbit IgG Alexa Flur 555. All assessments were performed in a blind manner.

### Electron microscopy

For scanning electron microscopy, kidney tissue were rinsed with PBS and postfixed with 1% aqueous osmium tetroxide for a total of 2.5 h. Subsequently, tissues were rinsed and dehydrated through a graded series of ethanol to absolute ethanol and critical point dried using liquid CO_2_. After mounting on stubs the specimens were sputter coated with a gold-palladium alloy for 1.5 min. Images were viewed with a Hitachi 2600 electron microscope.

For transmission electron microscopy, kidneys were immersed in cold modified Karnovsky's fixative containing 3% glutaraldehyde and 1% paraformaldehyde in sodium cacodylate buffer (pH 7.4) processed as follows^5^. After overnight fixation, kidneys were postfixed in 2% osmium tetraoxide, dehydrated through graded concentrations of ethanol and embedded in EMBed-812. Ultrathin sections of kidney were cut onto formvar-coated slot grids, and subsequently stained with uranyl acetate and lead citrate. The samples were viewed with a JEOL model 1200EX electron microscope (JEOL, Tokyo, Japan).

In another experiment, mouse tubular cells (MTCs) were grown on Thincerts (Greiner bio-one, Germany), and stimulated with crystals of CaOx (1,000 μg ml^−1^), MSU (500 μg ml^−1^), CPPD (500 μg ml^−1^) and cysteine (500 μg ml^−1^) for 24 h. Cells were then fixed for 1 h at room temperature by replacing the growth medium with 3% glutaraldehyde in PBS (pH 7.2). The Thincerts were then washed three times with PBS (pH 7.2) buffer and sliced into 3-mm squares. These pieces were postfixed in 2% aqueous osmium tetroxide for 2 h and then dehydrated through an ethanol series. After three washes in 100% ethanol, the pieces were transferred to acetone, then infiltrated and embedded in Epon. Sections ∼70 nm thick were cut using a diamond knife, collected on copper grids, and stained sequentially with uranyl acetate (saturated in 50% ethanol) and Reynolds' lead citrate. Sections were examined and photographed by a JEOL 1200 EXII transmission electron microscope at 80 kv.

For quick-freeze deep-etch electron microscopy, 1.5 mm bread slices of non-fixed kidney or kidney sections fixed overnight in 2% glutaraldehyde in 100 mM NaCl, 30 mM Hepes, 2 mM CaCl2, pH 7.2 (NaHCa) were used after rinsing in NaHCa. Tissues were then cooled to 4 °K with liquid helium mounted in a Balzers 400 vacuum evaporator fractured and etched for 2.5 min at −104 °C and rotary replicated with∼3 nm platinum. The process involved fracturing the kidney sample with a cryomicrotome under high vacuum, then allowed to partially freeze dry (this is called deep-etching), then covered with a thin film of platinum that forms a replica of the fractured and etched tissue. Then the sample was allowed to thaw so that the replica could be collected^40^ and viewed with transmission microscope (JEOL 1400 with attached AMT digital camera).

### RNA preparation and real-time quantitative PCR

Total RNA was isolated from kidneys using a Qiagen RNA extraction kit (Qiagen, Germany) following the manufacturer's instructions. After quantification RNA quality was assessed using agarose gels. From isolated RNA, complementary DNA was prepared using reverse transcriptase (Superscript II; Invitrogen, USA). Real-time reverse transcription PCR was performed using SYBRGreen PCR master mix and was analysed with a Light Cycler 480 (Roche, Germany). All gene expression values were normalized using 18 s RNA as a house keeping gene. All primers used for amplification were from Metabion (Martinsried, Germany). The expression of KIM-1, *π*-GST, TNFR1, RIPK1, RIPK3 and MLKL was tested using primers as follows: Kim-1: 5′-TCAGCTCGGGAATGCACA-3′ (for), 5′-TGGTTGCCTTCCGTGTCT-3′ (rev); *π*-GST: 5′-CGCAGCACTGAATCCGCACC-3′ (for), 5′-ACACCGCCCTCGAACTGGGAA-3′ (rev); TNFR1:-5′-GCAACAGCACCGCAGTAGCTGA-3′ (for), 5′-GTGCGTCCCTTGCAGCCACT-3′ (rev); RIPK1: 5′-GACTGTGTACCCTTACCTCCGA-3′ (for), CACTGCGATCATTCTCGTCCTG5′- (rev); RIPK3: 5′-GAAGACACGGCACTCCTTGGTA-3′ (for), 5′-CTTGAGGCAGTAGTTCTTGGTGG-3′ (rev), MLKL: 5′-CTGAGGGAACTGCTGGATAGAG-3′ (for), 5′-CGAGGAAACTGGAGCTGCTGAT-3′ (rev), Caspase-8: 5′-ATGGCTACGGTGAAGAACTGCG-3′ (for), 5′-TAGTTCACGCCAGTCAGGATGC-3′ (rev).

### Cell culture studies

MTCs, human synovial fibroblasts (K4IM cells) and L929 cells were maintained in DMEM/F12 (GIBCO, Invitrogen, CA, USA) containing 10% fetal calf serum, 1% penicillin–streptomycin. Human kidney cells (HK-2) were maintained in RPMI (GIBCO, Invitrogen, CA, USA) containing 10% fetal calf serum and 1% penicillin–streptomycin. MTCs are a cell line immortalized with SV-40 virus^41^, and K4IM cells are a cell line immortalized with SV-40 T large antigen^42^,^43^. MTCs and K4IM cells were generously provided by EG Neilson and PJ Nelson, respectively. L929 and HK-2 cells were originally purchased from ATCC, and were generously provided by B Luckow and PJ Nelson, respectively. Human renal progenitor cells were prepared from human kidney tissues using standard sieving technique through graded mesh screens (60, 80, 150 and 200 mesh)^24^. All cells were stimulated with different doses of crystals of CaOx (1–2 μm size; Alfa aesar, Germany), MSU (25–125 nm size; Invivogen, Germany), CPPD (25–125 nm size; Invivogen, Germany) and cystine (1–2 μm size; Sigma Aldrich, Germany) in different experiments. Cells were pretreated with necrostatin-1 (50 or 100 μM) or ZVAD-FMK (10 μM) or combination or RIPK3 inhibitor dabrafenib (Selleckchem, Germany) 30 min before crystals stimulations whenever required. Primary tubular epithelial cells were isolated from the kidneys and were maintained in DMEM/F12 containing 10% fetal calf serum, 1% penicillin–streptomycin, 125 ng ml^−1^ prostaglandin E1 (Calbiochem, Germany), 25 ng ml^−1^ EGF, 1.8 μg ml^−1^ l-thyroxine, 3.38 ng ml^−1^ hydrocortisone and 2.5 mg ml^−1^ of insulin–transferrin—sodium selenite supplement (all from Sigma Aldrich, Germany unless mentioned). Cells were grown to confluence before use in experiments. Primary renal human progenitor cells were plated in EGM-MV (Lonza Ltd., Basel, Switzerland) with 20% FBS (Hyclone, Logan, Utah). Generation of clones was achieved by limiting dilution in 96-well plates and in four-chamber glass slides (VWR International, West Chester, Pennsylvania). All the cells were cultured in an incubator at 37 °C, 5%CO_2_. CellTiter 96 non-radioactive cell proliferation assay (MTT) kit (Promega, Germany) was used to evaluate cell survival after 24 h following the manufacturer's instructions. Results were expressed as percentage of the vehicle control.

### Small interfering RNA experiments

MTCs were transiently transfected with 25 nM of SiRNA duplexes specific for RIPK3 (s80755), MLKL (s92952), Caspase-8 (s63394), and scrambled siRNA (negative control no.1) (Ambion, USA) using Lipofectamine RNAiMAX transfection reagent (Life Technologies, Germany). 24 h after transfection cells were challenged to CaOx (1,000 μg ml^−1^), MSU (500 μg ml^−1^), CPPD (500 μg ml^−1^) and cystine (500 μg ml^−1^) for 24 h. Cell death was measured by prodidium iodide positivity and cell viability was analysed using 3-(4,5-dimethylthiazol-2-yl)-2,5-diphenyltetrazolium bromide assay. Following small RNA sequences were used. *Ripk3*: 5′-CGGCUCUCGUCUUCAACAAtt-3′ (sense), 5′-UUGUUGAAGACGAGAGCCGgt-3′ (antisense); *Mlkl*: 5′-GAACCUGCCCGAUGACAUUtt-3′ (sense), 5′-AAUGUCAUCGGGCAGGUUCtt-3′ (antisense); *Casp8*: 5′-CAAGAACUAUAUUCCGGAUtt3′ (sense), 5′-AUCCGGAAUAUAGUUCUUGtg-3′ (antisense).

### Protein isolations and immunoblots

Protein from kidney tissues and cells were extracted using lysis buffer (Tris-HCl 50 mM, NaCl 150 mM, sodium orthovanadate 100 μM, sodium deoxycholate 0.5%, NP40 4%, Triton X-100 2%, EDTA 5 mM, sucrose 300 mM, and Roche protease inhibitors). Total cellular extracts were prepared in protein lysis buffer (Tris 10 mM, NaCl 10 mM, ethylenediaminetetraacetic acid 10 mM, HEPES 1 mol l^−1^, glycerol 20%, MgCl2 1 mol l^−1^, dithiothreitol 1 mol l^−1^, Triton 10% sodium fluoride 1 mol l^−1^ and Roche protease inhibitors). After determination of protein concentrations, 50 μg of the kidney protein or 10 μg of protein extracted from cells was mixed with 5 × sodium dodecyl sulfate loading buffer (100 mmol l^−1^ Tris-HCl, 4% sodium dodecyl sulfate, 20% glycerol and 0.2% bromophenol blue) for western blot analysis. Samples were heated at 95 °C for 5 min. Proteins were separated by sodium dodecyl sulfate-polyacrylamide gel electrophoresis and then transferred to a polyvinylidene difluoride membrane. Nonspecific binding to the membrane was blocked for 1 h at room temperature with 5% bovine serum albumin in tris-buffered saline buffer (20 mmol l^−1^ Tris-HCl, 150 mmol l^−1^ NaCl and 0.1% Tween 20). The membranes were then incubated overnight at 4 °C with the following primary antibodies: TNFR1 (1:500, Abcam, UK, #ab19139), RIPK1 (1:200, BD, Germany, #610459), RIPK3 (1:200, Abcam, UK, #ab152130), anti-human phospho-MLKL (1:200, Millipore, Germany, #ABC234), anti-human total MLKL (1:200, Millipore, Germany, #17-10400), total MLKL (1:500, Abcam, UK, #ab194699) and β-actin (1:1000, Cell Signalling, USA, #4967), followed by incubation with secondary antibody anti-biotin or anti-rabbit IgG labelled with HRP. Immunostained bands were detected using a chemiluminescence kit (ECL kit, GE Healthcare, UK). Immunoblot images in Figs 2 and 9 as well as Supplementary Figs 3 and 5 have been cropped for presentation. Full size images are presented in Supplementary Figs 9 and 10.

### Multi-parameter classification of cell death by flow cytometry

Cell death was induced in MTCs by irradiation with ultraviolet light type B at 1.5 mJ cm^−2^ s^−1^ or by treatment with CaOx crystals. Cell death was characterized by analysing six cytofluorometric parameters (size, granularity, penicillin–streptomycin exposure, plasma membrane integrity, mitochondrial membrane potential and DNA content) in a single tube measurement. This method detects eight phenotypically different subpopulations during the development of cell death *in vitro*. Briefly, collected cells were incubated for 30 min at room temperature with 400 μl of freshly prepared four-colour staining solution (1.8 μg ml^−1^ AxA5-FITC, 100 ng ml^−1^ prodidium iodide and 10 nM DiIC1(5), 1 ng ml^−1^ Hoechst 33342) in Ringer's solution and subsequently analysed. Flow cytometry was performed with a Gallios cytofluorometer (Beckman Coulter, Fullerton, USA). Excitation of FITC and prodidium iodide was at 488 nm, the FITC fluorescence was detected with the FL1 sensor (525/38 nm band pass (BP) filter), the prodidium iodide fluorescence with the FL3 sensor (620/30 nm BP), the DiIC1(5) fluorescence was excited at 638 nm and detected with the FL6 sensor (675/20 nm BP), and the Hoechst 33342 fluorescence was excited at 405 nm and detected with the FL9 sensor (430/40 nm BP). Electronic compensation was applied to reduce bleed through fluorescence. Data analysis was performed with Kaluza software version 2.0 (Beckman Coulter, Fullerton, USA). Cells were classified according to their location in the forward scatter (FSc; size) versus side scatter (SSc; granularity) dot plot and their staining patterns in the FL1 versus FL3 and FL6 versus FL9 dot plots as described elsewhere^44^. In brief, cells were considered viable, if they (V1) do not expose phosphatidylserine on their surfaces (V2) display high mitochondrial membrane potential and (V3), if they exclude propidium iodide. Apoptotic cells are defined by (A1) exposure on their surfaces of phosphatidylserine (A2) and exclusion of propidium iodide. Cells are considered necrotic, if they display (N) permeability for the propidium cation of their plasma membranes. Cells are considered necrotic, if they allow the penetration of propidium iodide. Primary necrotic cells (no signs of apoptosis) do not display nuclear hypochromicity for propidium iodide (nuclear DNA is preserved). If the nuclei show nuclear hypochromicity (apoptosis has already started) the cells were considered secondary necrotic (loss of membrane selectivity of a cell executing apoptosis).

### Statistical analysis

Data are presented as mean±s.e.m. A comparison of groups was performed using paired Students *t*-test or one-way analysis of variance with *post hoc* Bonferroni's correction was used for multiple comparisons. A value of *P*<0.05 was considered to indicate statistical significance.

## Additional information

**How to cite this article:** Mulay, S. R. *et al*. Cytotoxicity of crystals involves RIPK3-MLKL-mediated necroptosis. *Nat. Commun*. 7:10274 doi: 10.1038/ncomms10274 (2016).

## Supplementary Material

Supplementary Information Supplementary Figs 1-10

## References

[b1] Martinon F., Petrilli V., Mayor A., Tardivel A. & Tschopp J. Gout-associated uric acid crystals activate the NALP3 inflammasome. Nature 440, 237–241 (2006). 16407889 10.1038/nature04516

[b2] Hornung V. . Silica crystals and aluminum salts activate the NALP3 inflammasome through phagosomal destabilization. Nat. Immunol. 9, 847–856 (2008). 18604214 10.1038/ni.1631 PMC2834784

[b3] Dostert C. . Innate immune activation through Nalp3 inflammasome sensing of asbestos and silica. Science 320, 674–677 (2008). 18403674 10.1126/science.1156995 PMC2396588

[b4] Duewell P. . NLRP3 inflammasomes are required for atherogenesis and activated by cholesterol crystals. Nature 464, 1357–1361 (2010). 20428172 10.1038/nature08938 PMC2946640

[b5] Mulay S. R. . Calcium oxalate crystals induce renal inflammation by NLRP3-mediated IL-1beta secretion. J. Clin. Invest. 123, 236–246 (2013). 23221343 10.1172/JCI63679 PMC3533282

[b6] Schepers M. S., van Ballegooijen E. S., Bangma C. H. & Verkoelen C. F. Crystals cause acute necrotic cell death in renal proximal tubule cells, but not in collecting tubule cells. Kidney. Int. 68, 1543–1553 (2005). 16164631 10.1111/j.1523-1755.2005.00566.x

[b7] Schauer C. . Aggregated neutrophil extracellular traps limit inflammation by degrading cytokines and chemokines. Nat. Med. 20, 511–517 (2014). 24784231 10.1038/nm.3547

[b8] Vanden Berghe T., Linkermann A., Jouan-Lanhouet S., Walczak H. & Vandenabeele P. Regulated necrosis: the expanding network of non-apoptotic cell death pathways. Nat. Rev. Mol. Cell. Biol. 15, 135–147 (2014). 24452471 10.1038/nrm3737

[b9] Brinkmann V. . Neutrophil extracellular traps kill bacteria. Science 303, 1532–1535 (2004). 15001782 10.1126/science.1092385

[b10] Fink S. L. & Cookson B. T. Caspase-1-dependent pore formation during pyroptosis leads to osmotic lysis of infected host macrophages. Cell. Microbiol. 8, 1812–1825 (2006). 16824040 10.1111/j.1462-5822.2006.00751.x

[b11] Case C. L. . Caspase-11 stimulates rapid flagellin-independent pyroptosis in response to Legionella pneumophila. Proc. Natl Acad. Sci. USA 110, 1851–1856 (2013). 23307811 10.1073/pnas.1211521110 PMC3562791

[b12] von Moltke J., Ayres J. S., Kofoed E. M., Chavarria-Smith J. & Vance R. E. Recognition of bacteria by inflammasomes. Annu. Rev. Immunol. 31, 73–106 (2013). 23215645 10.1146/annurev-immunol-032712-095944

[b13] Linkermann A. & Green D. Mechanisms of disease: Necroptosis. N. Engl. J. Med. 370, 455–465 (2014). 24476434 10.1056/NEJMra1310050 PMC4035222

[b14] Wu J. . Mlkl knockout mice demonstrate the indispensable role of Mlkl in necroptosis. Cell Res. 23, 994–1006 (2013). 23835476 10.1038/cr.2013.91 PMC3731568

[b15] Zhang D. W. . RIP3, an energy metabolism regulator that switches TNF-induced cell death from apoptosis to necrosis. Science 325, 332–336 (2009). 19498109 10.1126/science.1172308

[b16] Linkermann A. . Two independent pathways of regulated necrosis mediate ischemia-reperfusion injury. Proc. Natl Acad. Sci. USA 110, 12024–12029 (2013). 23818611 10.1073/pnas.1305538110 PMC3718149

[b17] Dixon S. J. . Ferroptosis: an iron-dependent form of nonapoptotic cell death. Cell 149, 1060–1072 (2012). 22632970 10.1016/j.cell.2012.03.042 PMC3367386

[b18] Yang W. S. . Regulation of Ferroptotic Cancer Cell Death by GPX4. Cell 156, 317–331 (2014). 24439385 10.1016/j.cell.2013.12.010 PMC4076414

[b19] Degterev A. . Chemical inhibitor of nonapoptotic cell death with therapeutic potential for ischemic brain injury. Nat. Chem. Biol. 1, 112–119 (2005). 16408008 10.1038/nchembio711

[b20] Khan S. R. Nephrocalcinosis in animal models with and without stones. Urol. Res. 38, 429–438 (2010). 20658131 10.1007/s00240-010-0303-4 PMC2992101

[b21] Herlitz L. C., D'Agati V. D. & Markowitz G. S. Crystalline nephropathies. Arch. Pathol. Lab. Med. 136, 713–720 (2012). 22742545 10.5858/arpa.2011-0565-RA

[b22] Knauf F. . NALP3-mediated inflammation is a principal cause of progressive renal failure in oxalate nephropathy. Kidney Int. 84, 895–901 (2013). 23739234 10.1038/ki.2013.207 PMC3772982

[b23] Duprez L. . RIP kinase-dependent necrosis drives lethal systemic inflammatory response syndrome. Immunity 35, 908–918 (2011). 22195746 10.1016/j.immuni.2011.09.020

[b24] Angelotti M. L. . Characterization of renal progenitors committed toward tubular lineage and their regenerative potential in renal tubular injury. Stem Cells 30, 1714–1725 (2012). 22628275 10.1002/stem.1130

[b25] Chen X. . Translocation of mixed lineage kinase domain-like protein to plasma membrane leads to necrotic cell death. Cell Res. 24, 105–121 (2014). 24366341 10.1038/cr.2013.171 PMC3879712

[b26] Wang H. . Mixed lineage kinase domain-like protein MLKL causes necrotic membrane disruption upon phosphorylation by RIP3. Mol. Cell 54, 133–146 (2014). 24703947 10.1016/j.molcel.2014.03.003

[b27] Zhao J. . Mixed lineage kinase domain-like is a key receptor interacting protein 3 downstream component of TNF-induced necrosis. Proc. Natl Acad. Sci. USA 109, 5322–5327 (2012). 22421439 10.1073/pnas.1200012109 PMC3325682

[b28] Newton K. . Activity of protein kinase RIPK3 determines whether cells die by necroptosis or apoptosis. Science 343, 1357–1360 (2014). 24557836 10.1126/science.1249361

[b29] Linkermann A., Stockwell B. R., Krautwald S. & Anders H. J. Regulated cell death and inflammation: an auto-amplification loop causes organ failure. Nat. Rev. Immunol. 14, 759–767 (2014). 25324125 10.1038/nri3743

[b30] Lawlor K. E. . RIPK3 promotes cell death and NLRP3 inflammasome activation in the absence of MLKL. Nat. Commun. 6, 6282 (2015). 25693118 10.1038/ncomms7282 PMC4346630

[b31] Christofferson D. E., Li Y. & Yuan J. Control of life-or-death decisions by RIP1 kinase. Annu. Rev. Physiol. 76, 129–150 (2014). 24079414 10.1146/annurev-physiol-021113-170259

[b32] Ng G. . Receptor-independent, direct membrane binding leads to cell-surface lipid sorting and Syk kinase activation in dendritic cells. Immunity 29, 807–818 (2008). 18993083 10.1016/j.immuni.2008.09.013 PMC2642965

[b33] Neumann K. . Clec12a is an inhibitory receptor for uric acid crystals that regulates inflammation in response to cell death. Immunity 40, 389–399 (2014). 24631154 10.1016/j.immuni.2013.12.015

[b34] Orlowski G. M. . Multiple Cathepsins Promote Pro-IL-1beta Synthesis and NLRP3-Mediated IL-1beta Activation. J. Immunol. 195, 1685–1697 (2015). 26195813 10.4049/jimmunol.1500509 PMC4530060

[b35] McComb S. . Cathepsins limit macrophage necroptosis through cleavage of Rip1 kinase. J. Immunol. 192, 5671–5678 (2014). 24799565 10.4049/jimmunol.1303380

[b36] Baez S. An open cremaster muscle preparation for the study of blood vessels by *in vivo* microscopy. Microvasc. Res. 5, 384–394 (1973). 4709735 10.1016/0026-2862(73)90054-x

[b37] Mempel T. R., Moser C., Hutter J., Kuebler W. M. & Krombach F. Visualization of leukocyte transendothelial and interstitial migration using reflected light oblique transillumination in intravital video microscopy. J. Vasc. Res. 40, 435–441 (2003). 14530600 10.1159/000073902

[b38] Reichel C. A. . Chemokine receptors Ccr1, Ccr2, and Ccr5 mediate neutrophil migration to postischemic tissue. J. Leukoc. Biol. 79, 114–122 (2006). 16275892 10.1189/jlb.0605337

[b39] Yamaguchi S. . Study of a rat model for calcium oxalate crystal formation without severe renal damage in selected conditions. Int. J. Urol. 12, 290–298 (2005). 15828958 10.1111/j.1442-2042.2005.01038.x

[b40] Heuser J. Preparing biological samples for stereomicroscopy by the quick-freeze, deep-etch, rotary-replication technique. Methods Cell Biol. 22, 97–122 (1981). 6267417 10.1016/s0091-679x(08)61872-5

[b41] Haverty T. P. . Characterization of a renal tubular epithelial cell line which secretes the autologous target antigen of autoimmune experimental interstitial nephritis. J. Cell. Biol. 107, 1359–1368 (1988). 3170633 10.1083/jcb.107.4.1359 PMC2115243

[b42] Haas C., Aicher W. K., Dinkel A., Peter H. H. & Eibel H. Characterization of SV40T antigen immortalized human synovial fibroblasts: maintained expression patterns of EGR-1, HLA-DR and some surface receptors. Rheumatol. Int. 16, 241–247 (1997). 9106935 10.1007/BF01375656

[b43] Adams J. . 13-cis retinoic acid inhibits development and progression of chronic allograft nephropathy. Am. J. Pathol. 167, 285–298 (2005). 15972972 10.1016/S0002-9440(10)62973-2 PMC1603446

[b44] Munoz L. E. . Colourful death: six-parameter classification of cell death by flow cytometry--dead cells tell tales. Autoimmunity 46, 336–341 (2013). 23231469 10.3109/08916934.2012.755960

