# Supplementary material for: Cytotoxicity of crystals involves RIPK3-MLKL-mediated necroptosis
Source: Nat Commun. 2016 Jan 28;7:10274. doi: 10.1038/ncomms10274 (PMC4738349; doi:10.1038/ncomms10274)
Supplement: Supplementary Information — Supplementary Figs 1-10 [file ncomms10274-s1.pdf]

Supplementary figure 1

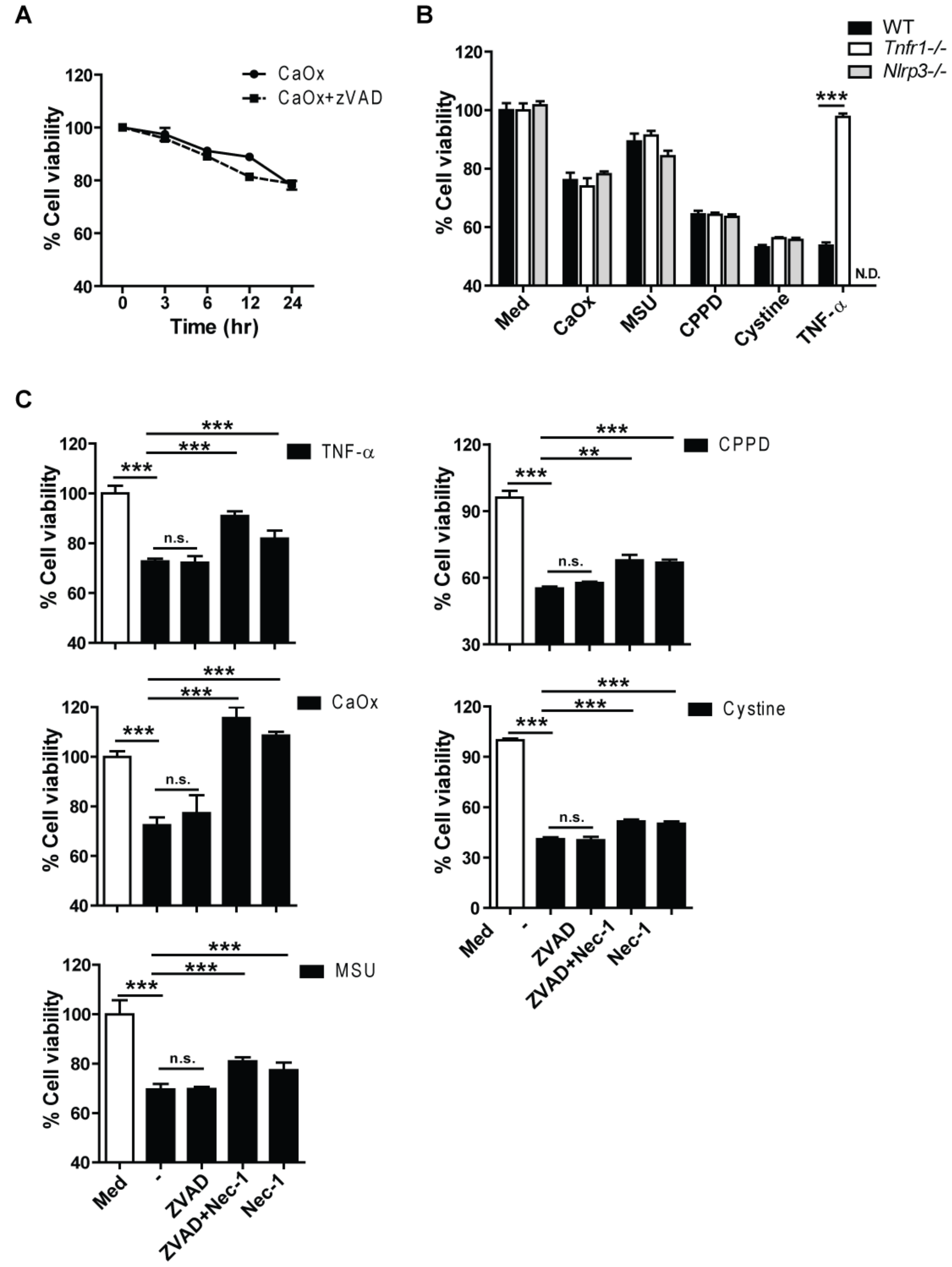

Supplementary fig. 1. Crystals induce necroptosis does not involve caspases, TNF receptor or NLRP3. A. Mouse tubular epithelial cells were pretreated with zVAD-FMK (10µM) and exposed to calcium oxalate (CaOx) crystals (1000µg/ml). Cell viability was determined by MTT assay. B. Primary tubular epithelial cells were isolated from wild-type (WT) or *Nlrp3*- or *Tnfr1*-deficient mice and exposed to crystals of calcium oxalate (CaOx) (1000µg/ml), monosodium urate (MSU) (500µg/ml), calcium pyrophosphate dehydrate (CPPD) (500µg/ml), cystine (500µg/ml) or recombinant TNF-α (300ng/ml) as positive control. Cell viability was determined by MTT assay 24h later. Note that neither lack of NLRP3 or TNFR1 affected crystal-induced cytotoxicity. C. Mouse tubular epithelial cells were pretreated with ZVAD-FMK (10µM), necrostatin (Nec)-1 (100µM) or combination and exposed to recombinant TNF-α (300ng/ml) as well as crystals of calcium oxalate (CaOx) (1000µg/ml), monosodium urate (MSU) (500µg/ml), calcium pyrophosphate dehydrate (CPPD) (500µg/ml) and cystine (500µg/ml). Cell viability was determined by MTT assay 24h later. Data are expressed as mean cell viability ± SEM of three independent experiments. Baseline viability is set as 100%. Data was analyzed using student's t test.* $p<0.05$, ** $p<0.01$, *** $p<0.001$ either versus medium control. N.D.= not done.

Supplementary figure 2

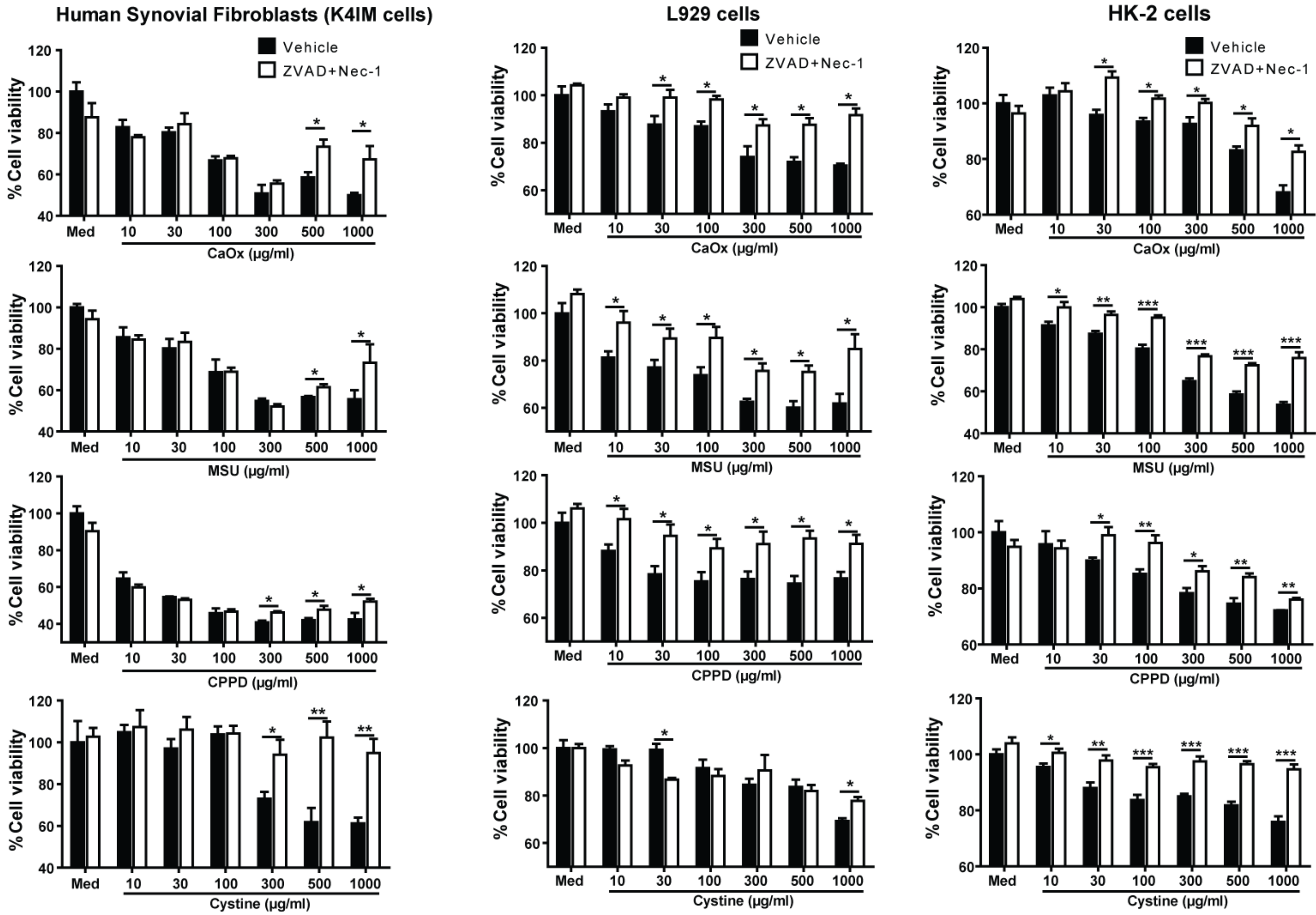

Supplementary fig. 2. Crystals induce necroptosis in human synovial fibroblasts, L929 and HK-2 cells. Human synovial fibroblasts, L929 cells, and HK-2 cells were all exposed to crystals of calcium oxalate (CaOx), monosodium urate (MSU), calcium pyrophosphate dehydrate (CPPD) and cystine at different concentrations as indicated in the presence or absence of ZVAD-FMK-FMK (10µM) and necrostatin (Nec)-1 (100µM). Cell viability was determined by MTT assay 24h later. Data are expressed as mean cell viability ± SEM of three independent experiments. Baseline viability is set as 100%. Data was analyzed using student's t test. * p<0.05, ** p<0.01, *** p<0.001 either versus vehicle control.

Supplementary figure 3

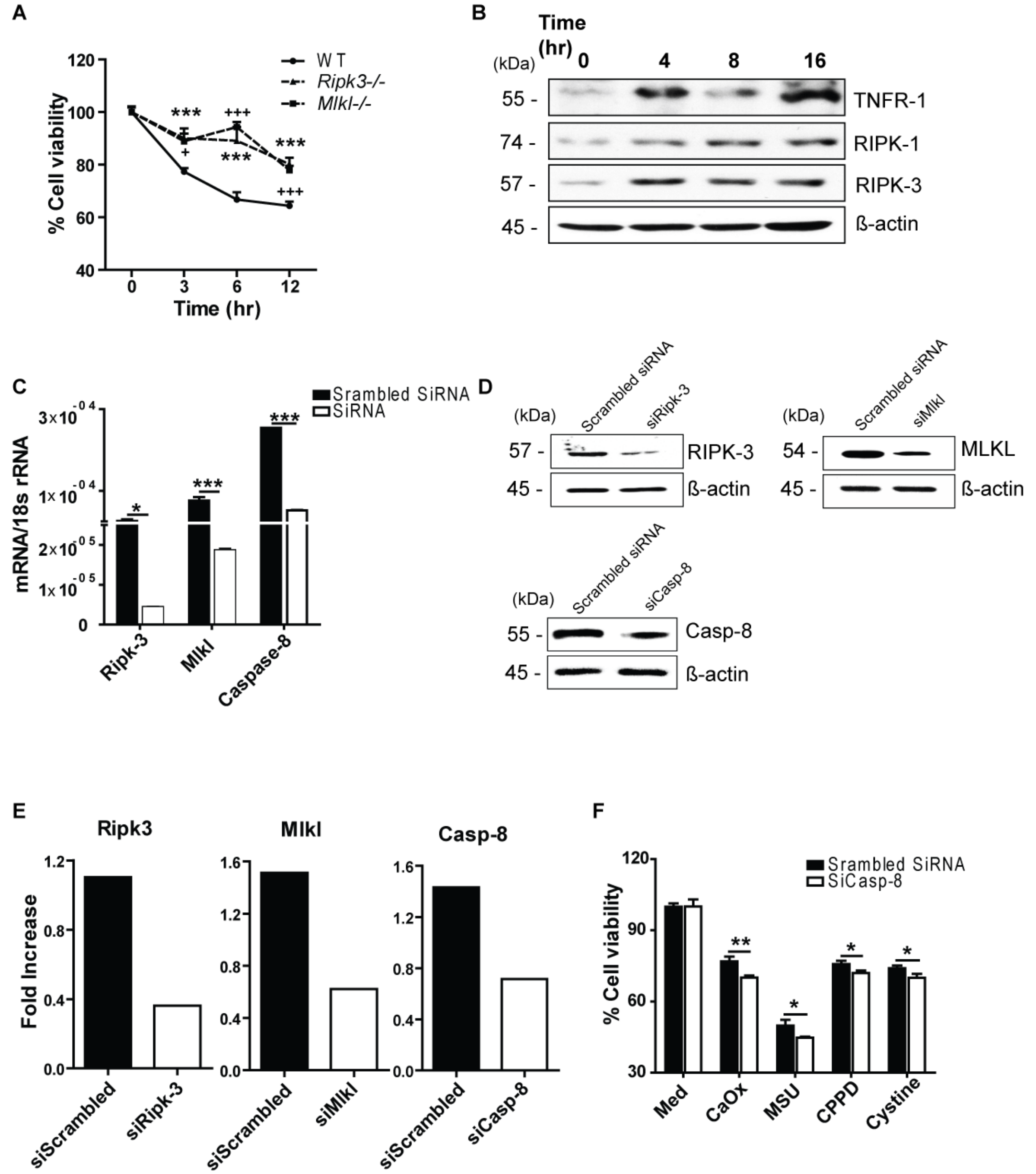

Supplementary fig. 3. Kinetics of calcium oxalate crystal-induced cell death *in vitro*. A: Primary tubular epithelial cells isolated from wild type, *Ripk3*-deficient, and *Mlkl*-deficient mice were exposed to calcium oxalate (1000µg/ml) for different times as indicated. B: Protein expression of TNFR1, RIPK1 and RIPK3 was determined by western blot from total proteins isolated at different times as indicated after stimulation of mouse tubular epithelial cells with crystals of calcium oxalate (1000µg/ml). β-actin was used as loading control. C-E: Tubular epithelial cells were transfected with siRNA specific for RIPK3, MLKL, Caspase-8, and control scrambled siRNA. The efficiency of knockdown was determined by RT-PCR for indicated genes (C), representative western blot (D) and their quantification (E). F: Mouse tubular epithelial cells were transfected with specific small inhibitor (si) RNA for Casp8, and a control siRNA of scrambled sequence before being exposed to crystals of (CaOx) (1000µg/ml), monosodium urate (MSU) (500µg/ml), calcium pyrophosphate dihydrate (CPPD) (500µg/ml) and cystine (500µg/ml). Cell viability was assessed by MTT assay. Data are expressed as mean ± SEM of three independent experiments. Data was analyzed using student's t test. * $p<0.05$, *** $p<0.001$ either versus control.

Supplementary figure 4

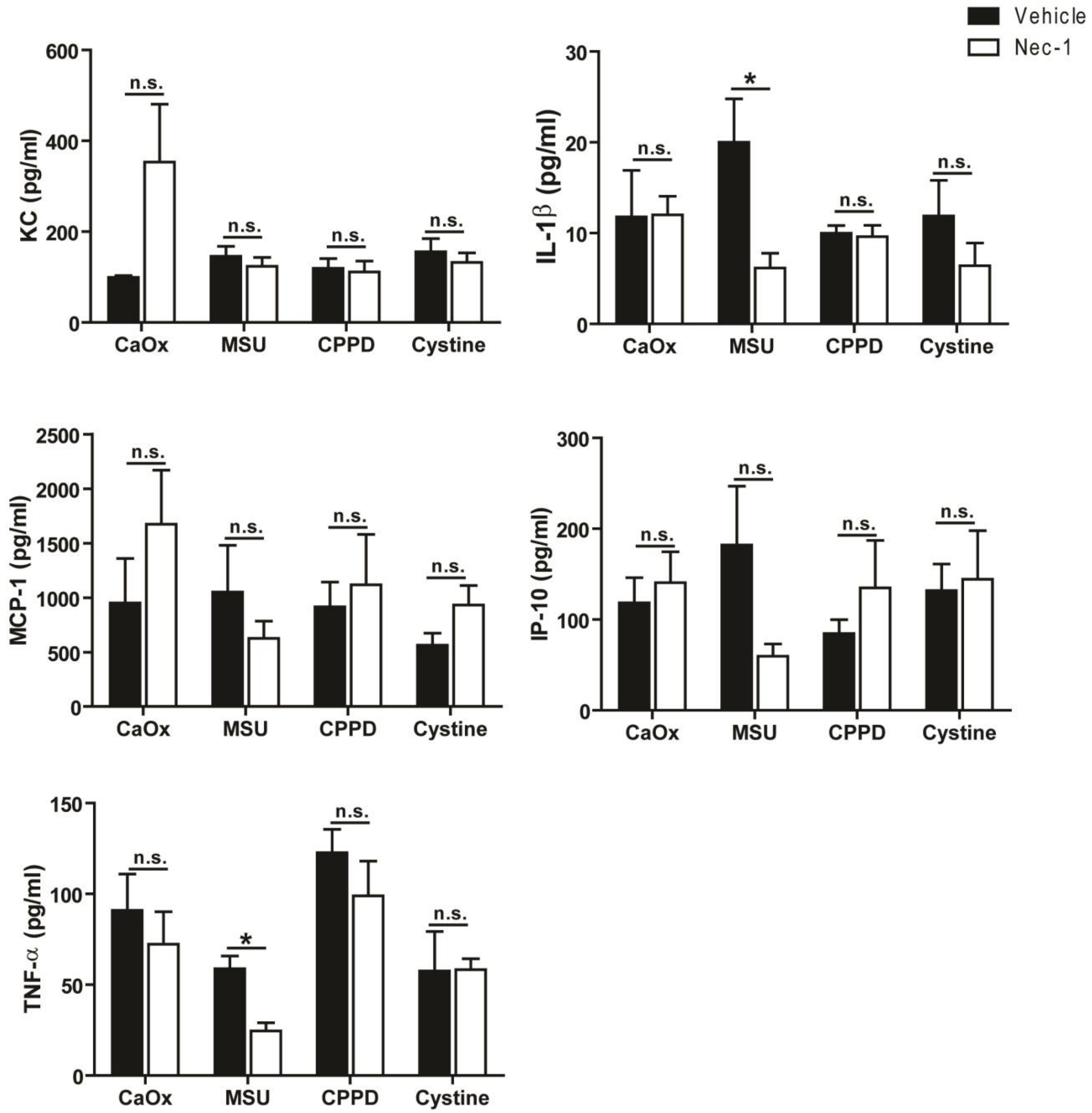

Supplementary fig. 4. Cytokines release in air pouch fluid after crystal injections. Crystals of calcium oxalate (CaOx), monosodium urate (MSU), calcium pyrophosphate dehydrate (CPPD) and Cystine (2.5mg) were injected in the air pouches of wild-type C57BL/6 mice treated either with vehicle or necrostain (Nec)-1 (1.65mg/kg *i.p.*). Data are cytokine ELISA results of air pouch lavage fluids taken 24h after crystal injection. Data was analyzed using one-way ANOVA with post-hoc Bonferroni`s correction. Data are means ± SEM of 5 mice in each group. * p<0.05 versus vehicle control. N.S. = not significant.

Supplementary figure 5

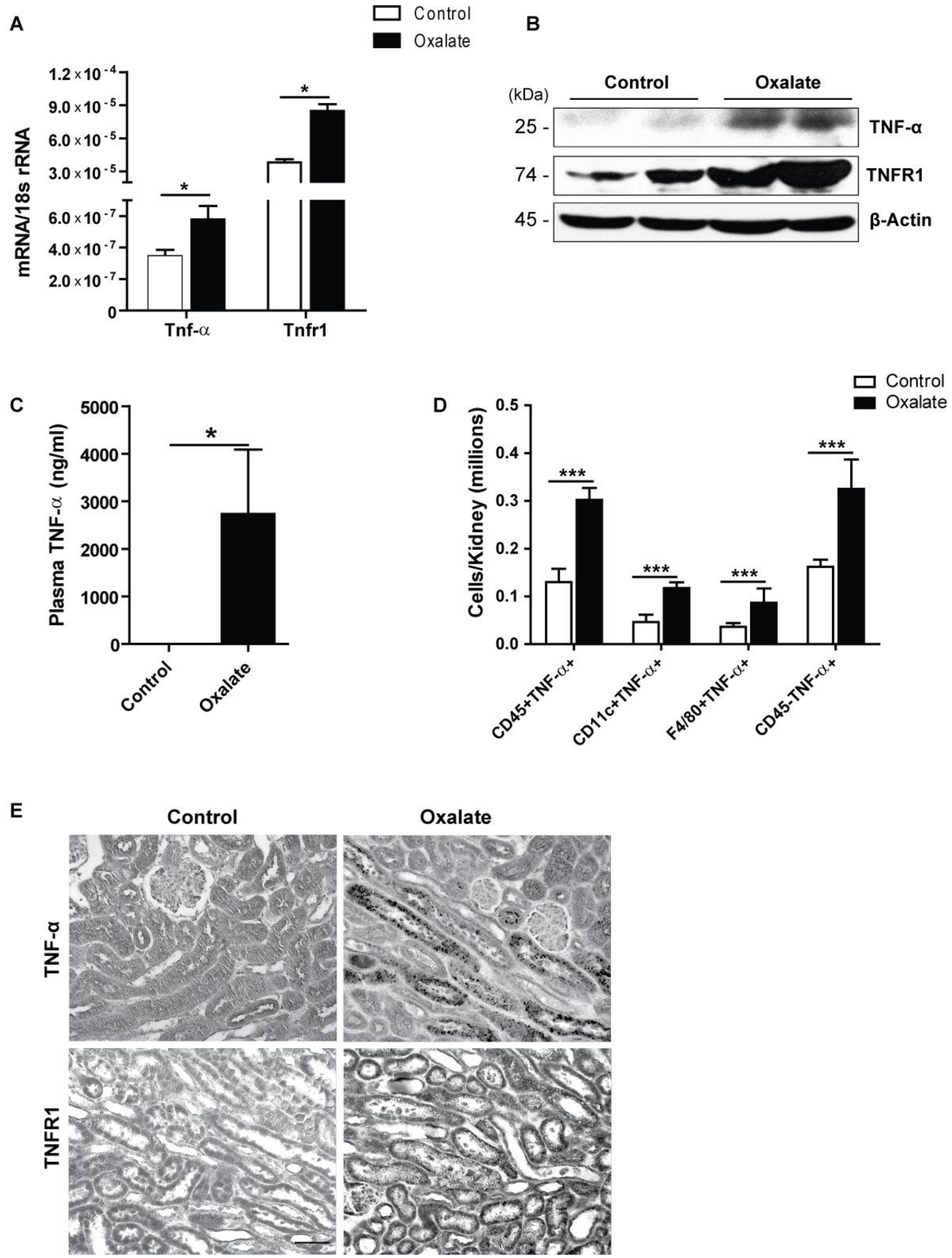

Supplementary fig. 5. Expression of TNF and TNF receptors in murine kidneys during acute oxalate nephropathy. A: Oxalate injection to wild-type C57BL/6 mice resulted in induction of TNF-α and TNFR1 kidney mRNA expression after 24h. B: Respective western blots are shown using β-actin as loading control. C: TNF-α levels in plasma of mice control and oxalate injected mice were analyzed by ELISA. D: Flow cytometry for intracellular TNF-α of kidney cell suspensions from the same experiment identifies various leukocytes subsets as well as CD45 positive renal parenchymal cells as a source of TNF-α protein expression. Quantitative data are means ± SEM of 5 mice in each group. Data was analyzed using student's t test. * $p<0.05$, *** $p<0.001$ either versus control. E: Immunostaining for TNF-α and TNFR1 revealed robust positivity of all of these markers in tubular epithelial cells in association with luminal crystal plugs. Original magnification of the representative images 200x. Scale bar 100µm.

Supplementary figure 6

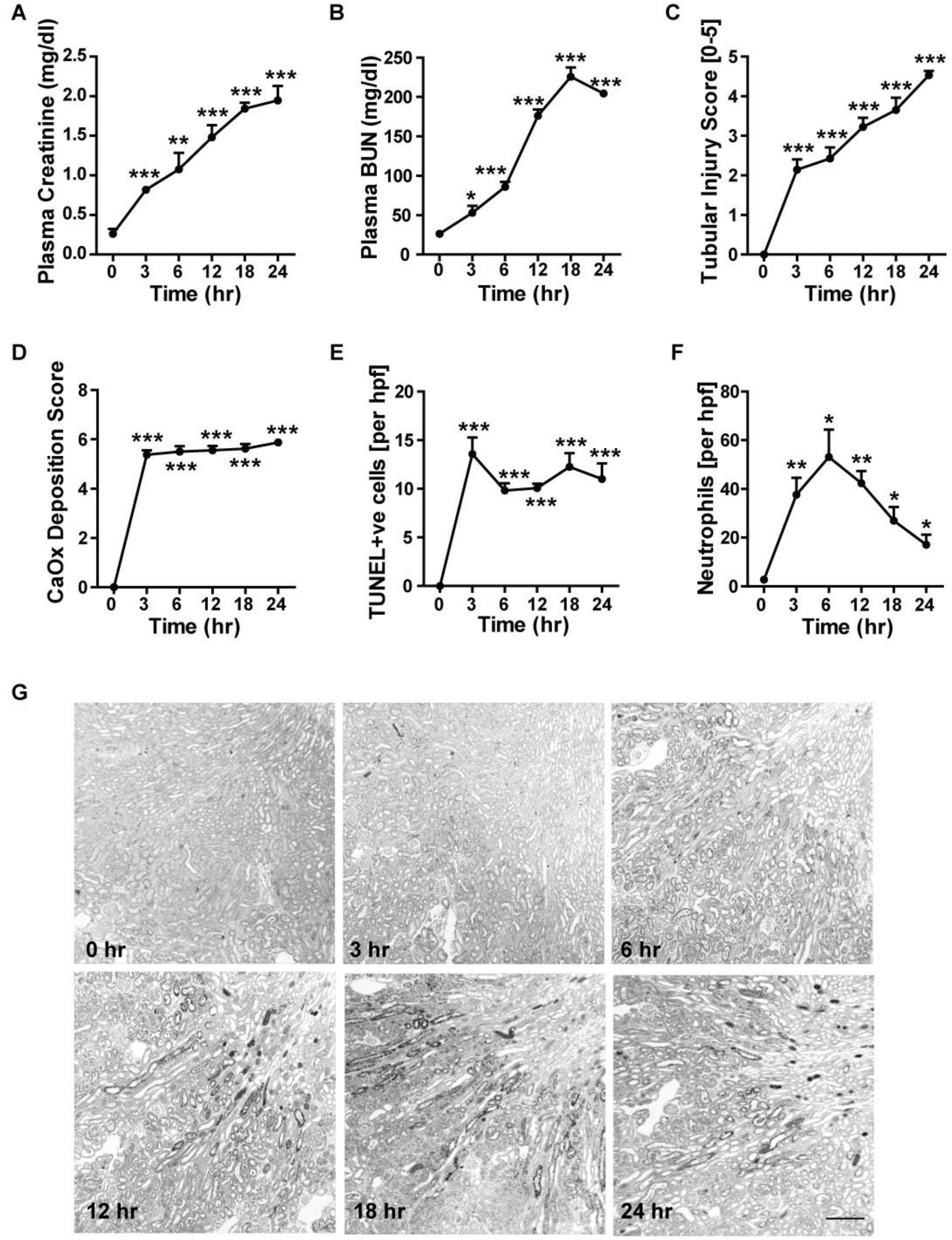

Supplementary fig. 6. Kinetics of acute oxalate nephropathy *in vivo*. C57BL/6 mice were sacrificed at different time points viz. 0, 3, 6, 12, 18 and 24 hrs after oxalate injection. A. Plasma creatinine. B. Plasma BUN. C. Tubular injury score. D. Calcium oxalate (CaOx) deposition score. E. $TUNEL^{+ve}$ dead cells. F. Neutrophils count. Quantitative data are expressed as mean ± SEM of 5 mice in each group. Data was analyzed using student's t test. * $p<0.05$, ** $p<0.01$, *** $p<0.001$ all versus vehicle control. G. Immunostaining for TNF-α. Original magnification od the representative images 100x. Scale bar 0.5mm.

Supplementary figure 7

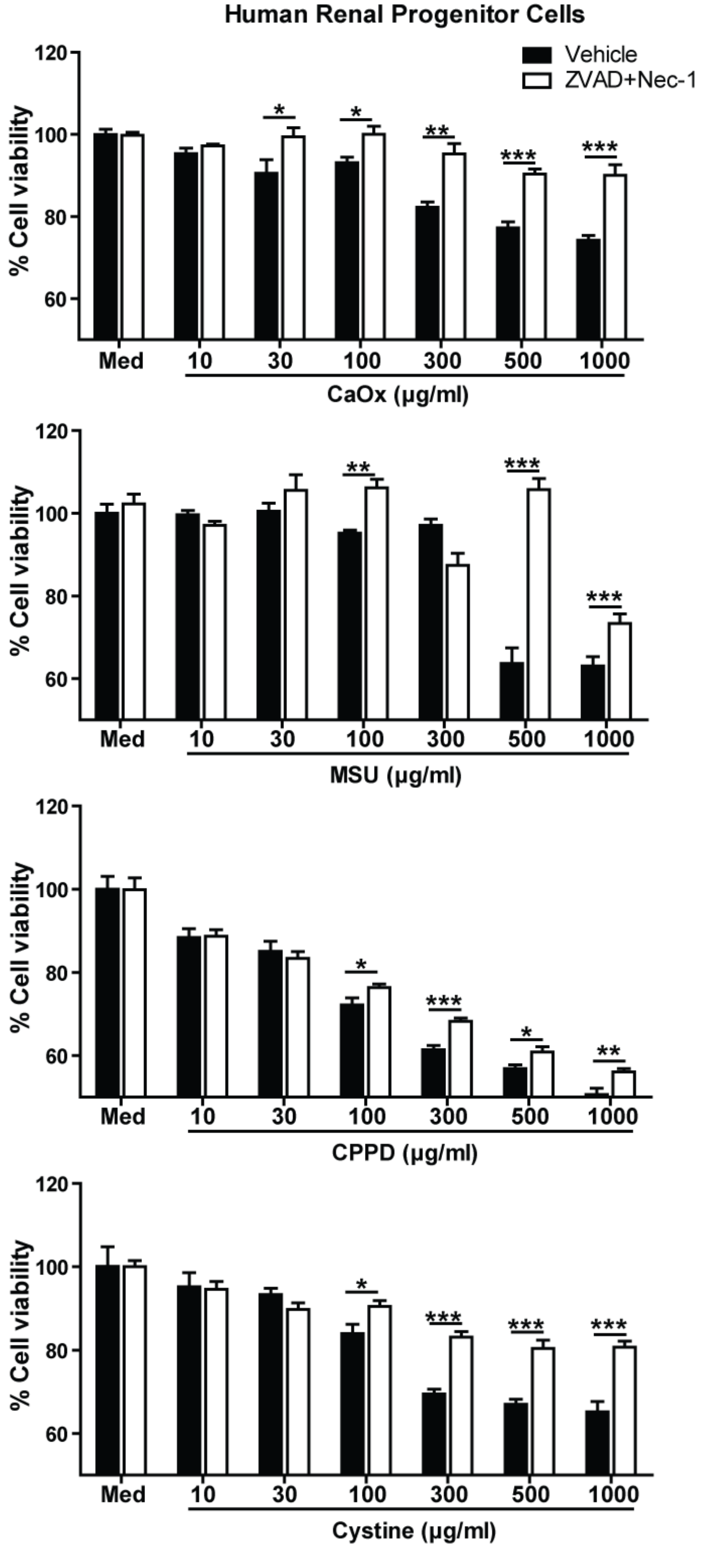

Supplementary fig. 7. Crystal induced necroptosis in human renal progenitor cells. Primary human renal progenitor cells were exposed to calcium oxalate (CaOx), monosodium urate (MSU), calcium pyrophosphate dehydrate (CPPD) and cystine at different concentrations as indicated in the presence or absence of ZVAD-FMK-FMK (10µM) and necrostatin (Nec)-1 (100µM). Cell viability was determined by MTT assay 24h later. Data are expressed as percentage cell viability ± SEM of three independent experiments. Baseline viability is set as 100%. Data was analyzed using student's t test. * $p<0.05$, ** $p<0.01$, *** $p<0.001$ all versus vehicle control.

Supplementary figure 8

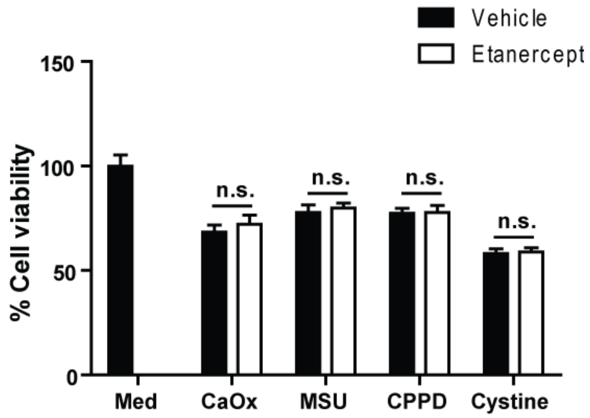

Supplementary fig. 8. Crystal induced necroptosis does not involve TNF-α. Tubular epithelial cells were pretreated with etanercept (100mg/ml) and exposed to crystals of calcium oxalate (CaOx) (1000µg/ml), monosodium urate (MSU) (500µg/ml), calcium pyrophosphate dehydrate (CPPD) (500µg/ml), and cystine (500µg/ml). Cell viability was determined by MTT assay 24h later. Data are expressed as mean cell viability ± SEM of three independent experiments. Baseline viability is set as 100%. Data was analyzed using student's t test. N.S. = not significant.

Supplementary Figure 9

Figure 2A

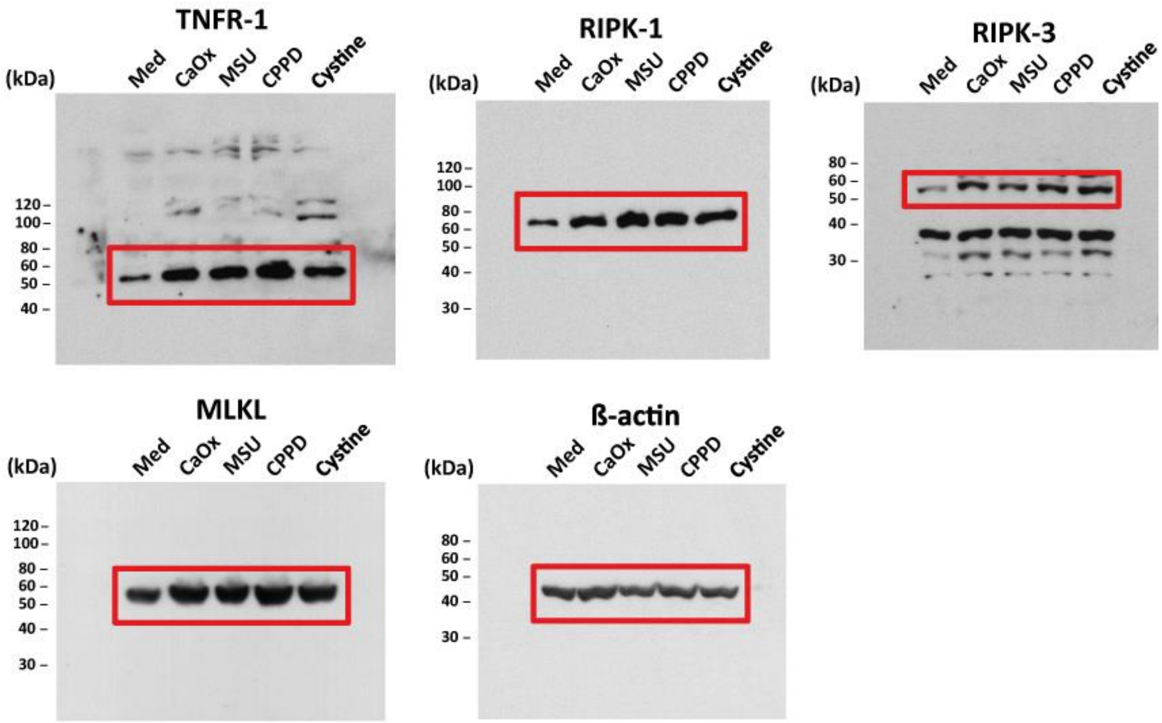

Figure 9D

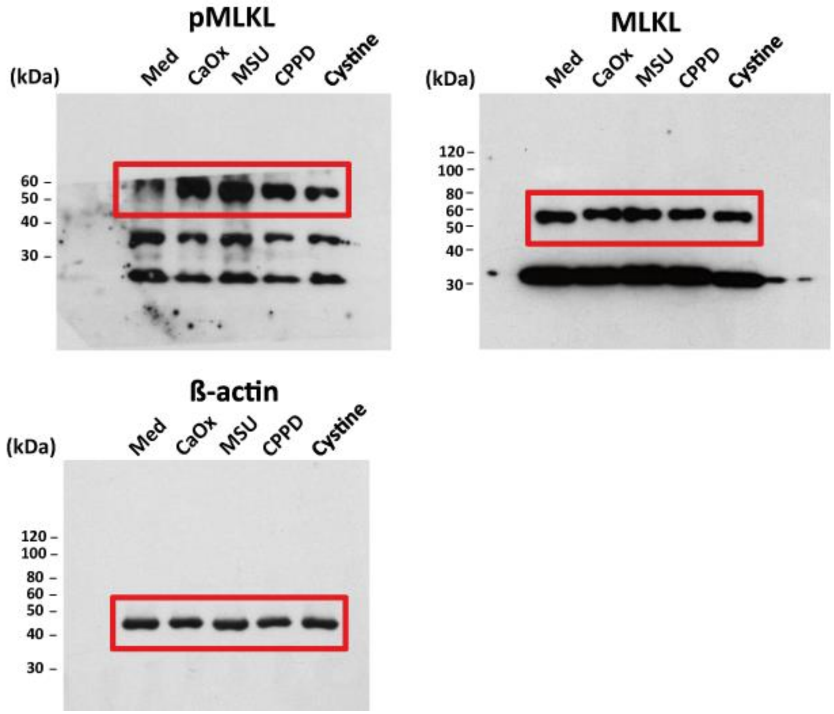

Supplementary fig. 9. Uncropped images of western blots. For figure 2A and 9D. Red boxes indicate the part of the blot used for cropped presentation.

Supplementary Figure 10

Supplementary Figure 3B

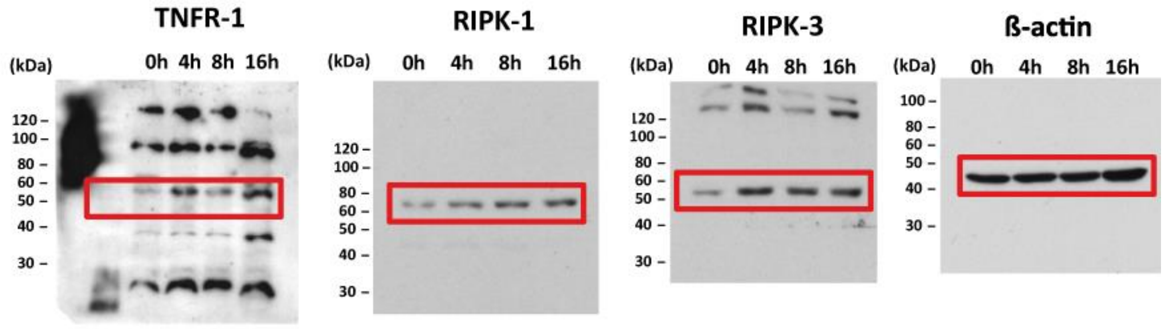

Supplementary Figure 3D

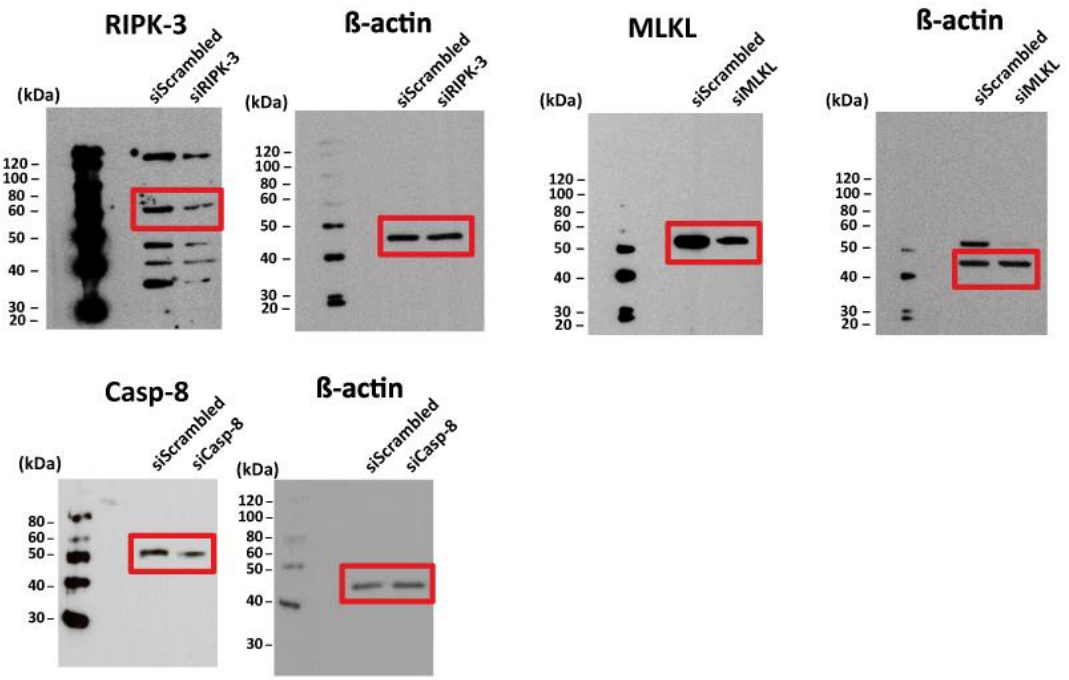

Supplementary Figure 5B

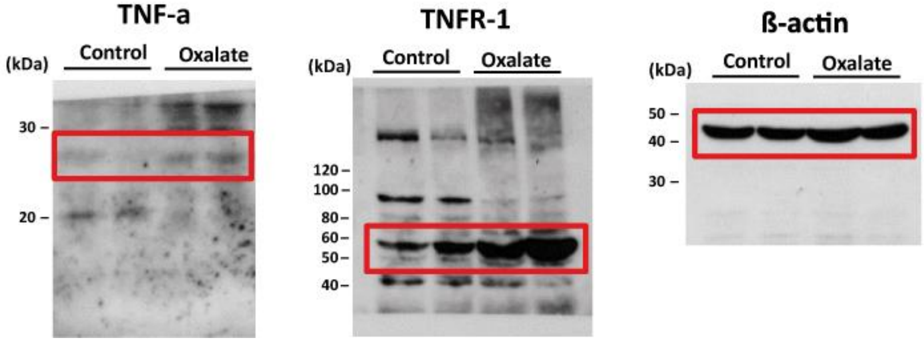

Supplementary fig. 10. Uncropped images of western blots. For supplementary figure 3B, 3D and 5B. Red boxes indicate the part of the blot used for cropped presentation.
